# Nanoparticle Synthesis and Their Integration into Polymer-Based Fibers for Biomedical Applications

**DOI:** 10.3390/biomedicines11071862

**Published:** 2023-06-29

**Authors:** Joana M. Domingues, Catarina S. Miranda, Natália C. Homem, Helena P. Felgueiras, Joana C. Antunes

**Affiliations:** 1 Centre for Textile Science and Technology (2C2T), Campus of Azurém, University of Minho, 4800-058 Guimarães, Portugal; joana.domingues@2c2t.uminho.pt (J.M.D.); catarina.miranda@2c2t.uminho.pt (C.S.M.); helena.felgueiras@2c2t.uminho.pt (H.P.F.); 2 Simoldes Plastics S.A., Rua Comendador António da Silva Rodrigues 165, 3720-193 Oliveira de Azeméis, Portugal; natalia.homem@simoldes.com; 3 Fibrenamics, Institute of Innovation on Fiber-Based Materials and Composites, Campus of Azurém, University of Minho, 4800-058 Guimarães, Portugal

**Keywords:** nanoparticles, polymer-based fibers, functionalization, bioactivity, biomedical applications

## Abstract

The potential of nanoparticles as effective drug delivery systems combined with the versatility of fibers has led to the development of new and improved strategies to help in the diagnosis and treatment of diseases. Nanoparticles have extraordinary characteristics that are helpful in several applications, including wound dressings, microbial balance approaches, tissue regeneration, and cancer treatment. Owing to their large surface area, tailor-ability, and persistent diameter, fibers are also used for wound dressings, tissue engineering, controlled drug delivery, and protective clothing. The combination of nanoparticles with fibers has the power to generate delivery systems that have enhanced performance over the individual architectures. This review aims at illustrating the main possibilities and trends of fibers functionalized with nanoparticles, focusing on inorganic and organic nanoparticles and polymer-based fibers. Emphasis on the recent progress in the fabrication procedures of several types of nanoparticles and in the description of the most used polymers to produce fibers has been undertaken, along with the bioactivity of such alliances in several biomedical applications. To finish, future perspectives of nanoparticles incorporated within polymer-based fibers for clinical use are presented and discussed, thus showcasing relevant paths to follow for enhanced success in the field.

## 1. Nanoparticles

Nanoscience and nanotechnology represent an expanding area, involving structures, devices, and systems with novel properties and functions. Nanotechnologies contribute to a wide panoply of scientific domains, including physics, material science, chemistry, biology, and engineering. Nanotechnology uses structures with controlled size and shape at the nanometer scale, and its novelty relies particularly on its ability to take advantage of some of the materials’ properties that are enhanced at the nanoscale [1]. In only a few decades, nanotechnology has become of fundamental importance to multiple industrial applications of which medical devices such as diagnostic biosensors, drug delivery systems, and imaging probes can be highlighted.

Nanoparticles (NPs) are a wide class of materials that include particulate substances, having sizes ranging from 1 to 100 nm [2]. This feature lends them large surface to volume ratio, making it possible to endow ordinary products with new functionalities [3,4]. In textiles, their multifunctionality may include self-cleaning, antimicrobial activity, decomposition of chemical agents, UV protection, antistatic, and flame retardancy properties, which can be very helpful for personal protective equipment (PPE) and biomedical applications, including wound dressings [5]. NPs play a key and significant role in this technological evolution since they show outstanding surface properties that allow their effect to be multiplied when compared with bulky traditional additives and materials. For example, nanomaterials are being used to build a new generation of solar cells, where tin oxide nanoparticle inks are used for printable perovskite solar cells to aid in the selective movement of electrons, which is a critical phase in the production of energy [6]; packaging in food industry where antimicrobial inorganic NPs are incorporated into food packaging to avoid foodborne pathogens contamination [7]; and in biomedicine for cancer treatment, gene delivery, medical implants, tissue engineering (to help in osteogenesis and vascularization), and for medical imaging [8,9]. NPs can be made of organic (e.g., lipidic and polymeric NPs) and inorganic (e.g., metallic NPs) materials depending on their application [4,10] and can present different morphologies, such as spherical and tubular dimensions, 0D, 1D, 2D, or 3D, and be positively or negatively charged with tunable chemical and physical properties [4,11]; thus raising their potential for applications in the biomedical field including for enhancing biological and mechanical properties, antibacterial effects, gene or drug delivery, fashioning biosensing devices, tissue engineering implants, and even in medical imaging [12,13,14,15,16,17]. Additionally, NP drug delivery systems (5–250 nm [18]) have the potential to improve the current disease therapies due to their ability to deliver drugs locally in the optimum dosage range, often resulting in increased therapeutic drug effectiveness, weakened side effects and improved patient compliance [4,18]. The development of hydrophilic NPs as drug carriers represents an important system for the intravenous administration of drugs [19].

However, NPs’ application in biomedical sciences still presents some drawbacks. The rapid clearance of circulating NPs during systemic delivery and their instability in biological environments remain critical issues, being caused by interactions with biological barriers and tunable NP-related parameters, such as composition, size, surface modifications, core properties, and targeting ligand functionalization [18]. To overcome this problem, significant investigation is being conducted. The development of hybrid composite scaffolds, which are able to maximize the biological effects of NPs, may minimize their associated drawbacks [20,21]. One of the most important strategies is to incorporate NPs into/onto polymer-based electrospun nanofibers, as these nanofibers are superb local delivery carriers with high porosity that can be tuned in diameter to influence cell behavior, namely cell attachment, proliferation, migration, and differentiation [22]. On another hand, fiber-based scaffolds functionalized with NPs are gaining much attention in tissue engineering, biomedicine, and controlled drug delivery [21,23]. These can serve as platforms to achieve a modulated, localized, and controlled delivery of the intended therapeutic agents [21,22]. Fibers reinforced with NPs with adequate biocompatibility and biodegradability present usefulness for tissue engineering and drug delivery/pharmaceutical applications [22].

### 1.1. Inorganic NPs

In the past decades, the design and fabrication of metal-based NPs have been improved, especially in those used for biomedical applications. These are synthesized from metals in nanometric sizes, and despite the diversity of metallic elements used to produce these types of NPs, the most frequently incorporated in polymer-based fibers designed for applications in biomedicine are silver (Ag), gold (Au), iron (Fe), zinc (Zn), magnesium (Mg), cerium (Ce), and titanium (Ti) [4]. Relevant advantages and limitations of inorganic NPs in the biomedical field are described in Table 1.

#### 1.1.1. Silver NPs

Among the several metal-based NPs, silver nanoparticles (AgNPs) have been extensively explored for their versatility and applicability, receiving special attention in a great variety of fields from chemistry to medicine [24]. These have superior physical, chemical, and biological characteristics compared to their bulk forms, and their properties (physical, optical, and catalytic) are influenced by their size, distribution, morphological shape, and surface features [24]. They are particularly attractive as antimicrobial and anticancer therapeutics, for water disinfection, medical diagnostics, and optoelectronics [24]. Therefore, physical, chemical, and optical properties of AgNPs are key factors in optimizing their use and should be considered during synthesis, namely size distribution, surface properties, particle composition, morphology, dissolution rate, and the type of capping/reducing agent used [24].

Nowadays, the AgNPs synthesis methodologies are categorized into physical, chemical, and biological. The physical synthesis of these NPs includes the evaporation-condensation and the laser ablation techniques [25]. The evaporation-condensation technique typically uses a gas phase route combined with a tube furnace to synthesize nanospheres at atmospheric pressure. The base metal source is evaporated into the carrier gas, allowing the final synthesis of the NPs [24]. With this method, spherical NPs with sizes ranging from 10 to 150 nm in diameter can be obtained [26,27]. Some authors concluded that reaction temperature and the geometric mean diameter of the size distribution of NPs are directly correlated, as well as particle concentration [26]. Another physical synthesis is laser ablation. After irradiating with a pulsed laser, the liquid environment only contains the AgNPs of the base metal source, cleared from other ions, compounds, or reducing agents, being considered a pure and uncontaminated synthesis approach [28]. Following this approach, the obtained average NP diameter is smaller than in the evaporation-condensation method, ranging from 2 to 20 nm [29,30], with the zeta potential varying between −33 and −68 mV [30].

The most common method to synthesize AgNPs is by chemical reduction of metal salts in aqueous colloidal dispersions or organic solvents [31]. In general, different reducing agents such as ascorbate, sodium citrate, sodium borohydride (NaBH_4_), elemental hydrogen, Tollen reagent, polyol process, poly (ethylene glycol)-block copolymers and N,N-dimethylformamide (DMF) can be used [32]. They act to reduce Ag+ leading to the formation of metallic silver (Ag0) that is followed by agglomeration into oligomeric clusters [32]. In addition, reducing and capping agents can easily be changed or modified to achieve the desired characteristics of AgNPs in terms of size distribution, shape, and dispersion rate. AgNPs can also be produced via polyol process yielding NPs of ≈3 and 5 nm in diameter. The obtained NPs were monodispersed and unaggregated [33].

Recently, the green chemistry metal NP synthesis method has been suggested as a valuable alternative to other synthesis methods whereby there are no requirements for reaction conditions, such as energy, temperature, and pressure, and no toxic chemicals are used. This type of synthesis employs microorganisms and plant extracts for NPs production. The biosynthesis of AgNPs by bacteria can occur via two processes: intracellular and extracellular. A non-enzymatic intracellular synthesis was reported in *Lactobacillus* A09, where Ag^+^ reduction occurred on the bacterial cell surface. The soluble Ag^+^ was reduced to the elemental Ag^0^ by an apparent redox route, in which the hydroxyl group of saccharides and the carboxylate anion of amino-acid residues located on the cell walls play a key role [34,35]. In case the biosynthesis of AgNPs occurs extracellularly, this is a very common example of how such a mechanism can be highlighted by using nicotinamide adenine dinucleotide (NAD) + hydrogen (H) (NADH) and NADH-dependent nitrate reductase enzyme to reduce Ag^+^ to metallic silver. Some authors produced AgNPs using the nitrate reductase that is present in *Bacillus licheniformis* with approximately 50 nm in size (X-ray powder diffraction and scanning electron microscopy (SEM) measurements) [35,36]. Due to their ability to produce larger amounts of AgNPs than bacteria, fungi are being considered for the biosynthesis of AgNPs [35,37]. Some microorganisms have shown the capacity to produce AgNPs intracellularly, where the intracellular components serve as both reducing and stabilizing agents, which is the case of the fungus *Verticillium species* that produces these NPs underneath its cell wall surface [38,39]. Despite these findings, the exact mechanism responsible for the synthesis of AgNPs by fungi is not yet well understood. Plant extracts have been widely used for AgNPs synthesis mainly because of their availability, safety, and low toxicity. In fact, plant extracts can act as both reducing and stabilizing agents during AgNPs biosynthesis, and due to the variability associated to the plant source, concentration, and combinations of phytochemicals, the NPs properties can be chosen by controlling the exact composition of the cocktail used to synthesize them [35,40]. A great variety of phytochemicals were identified as capable of producing AgNPs, including flavonoids, terpenes, terpenoids, flavones, phenolics, saponins, tannins, polysaccharides, and alkaloids [35,41]. Although there may be some differences in the mechanism of AgNPs synthesis trough plant extracts, it is believed that the reduction of Ag+ ions by specific functional groups is the main route. Sellami et al. reported the green synthesis of AgNPs using biological molecules of *Olea europaea* leaf extract, producing spherical, uniformly distributed and with an average size of 8 nm (transmission electron microscopy (TEM) measurements) [42].

#### 1.1.2. Gold NPs

Gold has gathered much interest in the research for nanomaterials due to its stability and low resistivity [43]. Gold nanoparticles (AuNPs) occur in the size ranges of 2 to 100 nm and the size can be controlled during their synthesis and functionalization with different groups [43]. These exhibit attractive intrinsic optical, physicochemical, and electronic properties that have been investigated for biomedical applications, such as drug delivery, photothermal therapy, biosensing, and theranostics [44,45]. Also, their high surface-to-volume ratio favors the improvement of biosensors sensibility [46,47]. Furthermore, AuNPs have great value as catalysts in chemical reactions and in agricultural crops since they are showing to be beneficial in seed germination, node elongation, and vegetative growth of plants, and in colorimetric sensing, detecting amino acids, peptides and proteins, nucleic acids, inorganic ions, and enzymes [48].

There are several methods to produce AuNPs, namely the Turkevich, Brust–Schiffrin, Martin method, green synthesis, and seed-mediated growth synthesis. The Turkevich method uses citric acid as the stabilizer agent to synthesize AuNPs, and in this method chloroauric acid is boiled and stirred and trisodium citrate dehydrate is added to obtain a colloidal suspension, reflecting the wine-red color characteristic of AuNPs [43]. Also, the diameter of the produced NPs can be modified by varying the amount of reactant used or by using different stabilizing factors [48]. The Brust–Schiffrin method is a two-phase synthesis that can generate thiolate-stabilized AuNPs. In this method, mercaptan reacts with thioalcohol and loses H atoms, which may result from the S-H bound oxidation and coupling with two adjacent Au atoms on the AuNP surface. AuNPs synthesized through this method have high thermal stability and air stability, no aggregation or decomposition occurs during repeated separation and redissolution, the size of AuNPs is easy to adjust and the dispersion is narrow, and it is relatively easy to functionalize and modify by ligand substitution. In the Martin method, NaBH_4_ is used as a reducing agent for the reduction of HAuCl_4_, whereas HCl and NaOH are used as stabilizing agents in this process to produce AuNPs as a colloidal dispersion. Interestingly, the NPs diameter can be tuned precisely from 3 to 5 nm, resulting in monodisperse AuNPs [48,49].

Similarly to the green synthesis of AgNPs, the green synthesis of AuNPs can be achieved through the presence of alkaloids, polyphenols, proteins, and other natural products in plant extracts, and the key role of their functional groups is reducing metals salts to zero-valent gold atoms and stabilizing NPs [50]. Recently, Zhang et al. synthesized AuNPs from *Euphorbia fischeriana* root, resulting in NPs with sizes ranging from 20 to 60 nm (High-Resolution (HR)-TEM measurements) [51]. Microorganisms, such as bacteria, have also been used for the biosynthesis of AuNPs. Recently a novel marine bacterium *Marinobacter algicola* was used to synthesize AuNPs, resulting in spherical NPs, with size ranging from 4 to 168 nm (TEM measurements) and a zeta potential of −31 mV [48,52]. Another way is exploring fungi to synthesize NPs, especially due to its scalability and cost-effectiveness [48]. Also, compared to other microorganisms, fungi can produce a larger number of extracellular enzymes capable of reducing metal salts to NPs. Some authors have synthesized AuNPs using an endophytic fungus *Fusarium solani* that has been isolated from the plant *Chonemorpha fragrans*. SEM analysis indicated that the average particle size was between 40 and 45 nm [48,53]. The process of seed-mediated synthesis of AuNPs can be divided in two main steps. In the first step, a small-sized seed of AuNP is prepared. Secondly, HAuCl_4_ is present in the growth solution of the seed and acts as a stabilizer and reducing agent. The size, shape, and surface properties of the AuNPs seeds during seed-mediated growth synthesis are determined by the dosage and properties of reductants and stabilizers and their ratio to Au precursor [43]. Due to the diverse characteristics presented by AuNPs, they can be applied in more fields. Wei et al. produced gold nanorods capped with hexadecyltrimethylammonium bromide (CTAB), using NaBH_4_ as a reducing agent to reduce HAuCl_4_, followed by growth in a solution containing NaOH, HAuCl_4_, AgNO_3_, and HCl and reached an average diameter ranging from 20 to 60 nm (TEM measurements) [54].

#### 1.1.3. Iron Oxide NPs

Generally, iron oxides are prevalent in nature playing an essential role in many biological and geological processes and are widely used because of their low cost [55]. Iron oxide nanoparticles (IONPs) possess unique properties including superparamagnetic and high magnetic susceptibility, displaying aggregation behavior under a magnetic field [56]. Moreover, these NPs have a good colloidal stability and biocompatibility, making them suitable for biomedical applications, such as in diagnostics, imaging, magnetic separation, hyperthermia, cell proliferation, tissue repair, and drug delivery [56]. Also, they are very interesting to remove heavy metals from polluted water, due to their magnetic properties, large surface area, and reduced size [57]. In the past decades, much research has been accomplished to develop iron oxide NPs of tunable size, being also efficient and stable.

The synthesis methods of iron oxide NPs can be divided into physical, chemical, and biological [58]. In what concerns the physical ones, a widely used method is ball milling, which consists of a solid-state mechanical size reduction that converts iron precursors into iron oxide NPs inside a stainless-steel container filled with grinding micron-sized spheres, resulting in average particle sizes of less than 150 nm [58,59]. Another physical method is called electron beam deposition, where an electron beam is emitted towards a bulk high-purity iron material. The NPs are obtained through evaporation of the initial iron precursors on the patterned resist, accompanied by a lift-off process to remove the resist [58]. Kurapov et al. produced iron NPs by electron beam vapor deposition in a porous NaCl matrix. The synthesized NPs displayed an average particle size of 5–70 nm (SEM, TEM, and dynamic light scattering (DLS) measurements) [59]. Similarly, laser ablation is a physical method to synthesize iron oxide NPs where a solid target material is placed under a thin layer and is irradiated with a laser beam [58]. The most widely used lasers to perform this method comprehend: Titanium-doped sapphire (Ti:Sapphire), neodymium-doped yttrium aluminum garnet (Nd:YAG), and copper vapor lasers [58,60]. This method allows the production of spherical to hexagonal NPs with relatively uniform averaged diameters of around 15 nm [61]. Another method is known as iron sputtering, in which the bulk material is vaporized through sputtering with a beam of inert gas ions [58,60]. In this method, the composition of the sputtered material remains the same as the target material; however, the type of sputtering gas employed can affect the NPs’ surface morphology, texture, and optical properties [60]. Tantalum (Ta) NPs were produced through sputtering in the presence of 2-butanol, heptane, and m-xylene for catalytic applications. Ta-heptane and Ta-xylene NPs actively promoted the oxygen reduction reaction, which is a very important process occurring at the cathode in fuel cells [62]. On another hand, spray pyrolysis is cost-effective, scalable, and consists in the delivery of NP precursors (in vapor state) into a hot reactor leading to the formation of small droplets inside the reactor [58,60]. This method allows the production of iron core-gold shell NPs, spherical and with average sizes of 260–390 nm [63]. According to the literature, the most commonly used chemical methods for iron oxide NPs synthesis include: co-precipitation, thermodecomposition, sol-gel, and microemulsion [58,64,65].

In addition to the physical methods, there are also chemical methods to produce IONPs. One of the most widely used and simplest chemical methods to synthesize IONPs is co-precipitation. In the co-precipitation method, ferrous and ferric salts are mixed stoichiometrically in an aqueous medium to generate iron oxide nanocrystals by precipitation of the ferrous and ferric ions in an alkaline environment. This method is often performed in the presence of hydrophilic polymers, such as dextran or starch, which bind to the iron oxide and form a hydrophilic and biocompatible surface, being considered simple, cost-effective and suitable for large-scale purposes [66]. However, the selected NPs produced through this method may contain several nanocrystals and a not controlled amount of the coating polymer, so the need to control the size distribution of nanocrystals has led to the development of the thermodecomposition method. The latter consists of the decomposition of iron pentacarbonyl, iron oleate, or iron acetylacetonate in organic solvents at high temperature, generating the “Fe-O” species in a controlled manner [67,68]. These monomers will either nucleate to form new nanocrystals or add to the surface of the existing nanocrystals depending on its concentration [69]. Researchers have synthesized monodispersed magnetite NPs by a general decomposition approach involving a high-temperature solution–phase reaction of Fe(acac) in the presence of phenyl ether with alcohol, oleic acid, and oleylamine, yielding NPs with 3 to 20 nm in diameter (TEM measurements) and inverse spinel structure [70,71]. Another effective process to produce IONPs is the sol-gel technique. It involves a hydrolysis of the NPs precursors, usually metal alkoxides in the presence of water or alcohols, followed by a condensation process where metal oxide linkages are established. The last steps involve the drying of the gel or a heat treatment to obtain the NPs [58,72]. Lopez et al. produced ultra-small IONPs trough a microwave assisted sol-gel method by heating (210 °C—30 min) iron (III) acetylacetonate in the presence of benzyl alcohol yielding NPs of 6 nm in size (TEM measurements) [73,74]. The microemulsion process requires a thermodynamically stable and isotropic dispersion containing a polar phase, a non-polar phase, and a surfactant that will serve as a nano-reactor providing the adequate environment for the nucleation and controlled growth of NPs [58,64]. The most widely employed amphiphilic surfactants for microemulsion systems are dioctyl sodium dodecyl sulfate (DSS), cetyltrimethylammonium bromide (CTAB), sodium dodecyl sulfate (SDS), and Tween 20 or Tween 80 [64]. Salvador, M. et al. produced superparamagnetic IONPs via microemulsion, using CTAB as surfactant, resulting in droplets of around 0.3 and 0.5 µm (TEM measurements) [75].

#### 1.1.4. Zinc Oxide NPs

Zinc oxide nanoparticles (ZnO NPs) are considered one of the most relevant metal oxide NPs due to their physical and chemical characteristics, currently being employed in several fields [76,77,78]. Zinc is present in all body tissues, and it is the main component in several enzymatic systems. In addition, zinc is very important in the body’s metabolism and in the synthesis of proteins and nucleic acids [76,77,78,79]. Zinc oxide (ZnO) is considered a generally recognized as safe (GRAS) substance by the Food and Drug Administration (FDA) agency, and nanosized ZnO is commonly applied as a food additive [80]. These factors have resulted in ZnO NPs becoming more of a target for biomedical investigation, such as drug delivery, anticancer, antibacterial, wound healing, and bioimaging [76,81,82]. Furthermore, ZnO NPs have optical, electrical, and photocatalytic properties, being applied in solar cells, photocatalytic processes, and as chemical sensors [75]. In the recent years, the methods to produce stable ZnO NPs have evolved, including several methods like sol-gel, chemical precipitation, solid-state pyrolytic, solution-free, and biosynthesis [76]. Sol-gel is a low-cost and simple approach that involves three steps: preparation of zinc precursor and ZnO clusters, and the crystal growth [83]. In the first step, a hygroscopic mixture of zinc acetate is obtained that is diluted with lithium hydroxide powder in the second step. The final step consist of a self-induced ZnO crystal growth [76,83]. Porous Zn-based and ZnO composites were successfully produced via a sol-gel process, using hexane as the drying solvent, resulting in microporous (>50 nm) flower-like microstructures with an average size of 23.2 nm [84]. The chemical precipitation method is the most used to synthesize ZnO NPs due to its simplicity and scalability [76]. Typically, a precipitator is added to a zinc precursor and mixed. Then, after a complete dissolution, zinc hydroxide is obtained and converted to ZnO through a sintering process at high temperatures [76,85]. Mahmood, N. et al. synthesized ZnO NPs using the oxalate co-precipitation method after calcination at 700 °C. Zine sulfate was used as a zinc soluble source and oxalic acid as a catalyst. The field emission scanning electron microscopy (FESEM) results indicated particles presented an average size of 80 nm [86]. The solid-state pyrolytic method is a low-cost and simple method with the advantage of producing high-quality ZnO NPs with controllable sizes [76]. The synthesis typically involves the use of zinc acetate and sodium bicarbonate to obtain a mixture that is pyrolyzed at the reaction temperature. The choice of the pyrolytic temperature can influence the particle sizes [76,87]. Some authors produced ZnO NPs via a rapid and highly efficient solid-state strategy, resulting in a hexagonal Wurtzite structure of NPs with an average diameter of 37.5 nm [88]. An also cost-effective method to produce ZnO NPs is the solution-free mechanochemical that is made of two major steps [89]. In the first step, zinc acetate and oxalic acid are mixed and grinded to obtain zinc oxalate NPs. The second step involves a process of thermal decomposition at a very high temperature to obtain ZnO NPs [76,89]. Recently, researchers have presented a solvent-free mechanochemical synthesis of ZnO NPs from ε-Zn(OH)_2_ crystals via high-energy ball milling, producing uniform ZnO NPs with sizes ranging from 10 to 30 nm (TEM and DLS measurements) [90]. Nowadays, the development of green chemistry to synthesize ZnO NPs has attracted more attention because it is environmentally friendly [91]. There is a broad variety of plants that can be used for the biosynthesis of ZnO NPs, such as the leaf of *Cochlospermum religiosum* (L.), *Azadirachta indica* (L.), *Plectranthus amboinicus*, *Andrographis paniculate*, *Aloe barbadensis*, the root extract of *Polygala tenuifolia*, the peel of *rambutan* (*Nephelium lappaceum* L), the rhizome extract of *Zingiber officinale*, the flower extract of *Trifolium pratense*, *Jacaranda mimosifolia*, the seeds of *Physalis alkekengi* L, among others [76,91,92,93,94,95,96,97,98,99,100,101]. Researchers have also prepared ZnO NPs from aqueous fruit extracts of *Myristica fragrans*, resulting in hexagonal wurtzite shape NPs with 66 nm of diameter and −22.1 mV of zeta potential, indicated by DLS data [102].

#### 1.1.5. Magnesium Oxide NPs

Magnesium oxide nanoparticles (MgONPs) have unique properties compared to bulk materials, such as high chemical stability, high electrical permittivity, high photocatalytic activity, and non-toxicity, making them an excellent candidate for medicine, agriculture, information technology, energy, electronics, and environmental applications [103]. Several approaches can be used to synthesize MgONPs, the most common being the sol-gel, combustion, solvo-/hydrothermal, co-precipitation, and green synthesis [103]. The sol-gel method is one of the most popular approaches used to fabricate MgONPs. It resorts to metal alkoxides together with the adequate solvents and reactants to form an homogenous solution that leads to crystal growth [103]. Magnesium acetate, magnesium nitrate, and magnesium methoxide are widely used as precursors for this process [103,104,105,106]. In a study published by Salman et al., MgONPs were prepared by a sol-gel approach in the presence of magnesium nitrate and sodium hydroxide. The fabricated MgONPs were crystalline with a spherical shape and a grain size of about 50 nm (FESEM measurements) [107]. The combustion method is frequently used for the production of these NPs, because of its efficiency and low cost [108]. It can be divided into two approaches, the self-propagating synthesis, and the volume combustion synthesis [108]. The self-propagating synthesis consists in spontaneous redox reactions ignited by an external source that takes place between the oxidizer (precursor) and the fuel (reductant) mixed in solution, resulting in the formation of solid products [109]. In the volume combustion synthesis, the sample with the oxidizer and the fuel is heated until the reaction is initiated, being more difficult to control [110]. Tharani et al., produced MgONPs by a simple combustion method using magnesium nitrate as the oxidizer and citric acid as the fuel. The MgONPs showed to have valuable optical properties, flakes-like structures, and flower-shaped morphology and average crystalline sizes of 20, 25, and 35 nm [111]. In the solvo-hydrothermal method, a precursor and a suitable solvent are placed in an autoclave and exposed to high temperature and pressure, resulting in the formation of the desired products [103]. It is this temperature and pressure exposure that causes the formation of materials carrying high crystallinity. It has been reported the use of magnesium nitrate hexahydrate and magnesium acetate as precursors and sodium hydroxide and urea as solvents, respectively [112,113]. Duong et al. produced MgO nanoplates through the hydrothermal calcination method with a diameter ranging from 40 to 60 nm (SEM measurements) and an average thickness of 5 nm [114]. The co-precipitation method is widely used in the formation of MgONPs, and it is based on the principle of precipitation, involving a liquid-phase synthesis [115]. The basic principle is the homogenization of the precipitation reaction involving nucleation and nuclei growth [103,116]. For this type of synthesis, the most commonly used precipitating agent is sodium hydroxide [103]. Frantina et al. produced MgO through co-precipitation method by calcination of magnesium carbonate. The obtained MgONPs were spherical in shape with an average particle size of 50.9 nm (SEM measurements) [117]. Another alternative approach to create MgONPs is the green synthesis where plant extracts, bacterial strains, enzymes, and vitamins can be used [103,118]. The extract of *Nephelium lappaceum* L., *Trigonella foenum-graecum, Tecomas tans* L., *Moringa oleifera, Swertia chirayaita, Saussurea costus*, *Dalbergia *sissoo, Rosmarinus officinalis** L., and *Rosa floribunda* powder have been used to produce MgONPs through the green approach [103,119,120,121,122,123,124,125,126]. Recently, Kumar et al. produced MgO NPs using *Camellia sinensis* tea leaves extract as a reducing agent. The XRD pattern indicated that the produced MgONPs had a cubic structure and the SEM measurements indicated that the size of NPs was in the range of approximately 65 nm [127].

#### 1.1.6. Cerium Oxide NPs

Cerium is a rare earth metal, existing in both CeO_2_ and Ce_2_O_3_ in bulk state [128]. Cerium oxide nanoparticles (CeNPs) exhibit great antioxidant properties due to the self-regeneration of their surface, that is based on redox-cycling between 3^+^ and 4^+^ states for cerium [129]. These NPs can be used in several fields, ranging from engineering to biology, such as solid-oxide fuel cells, protection materials, high-temperature oxidation, solar cells, drug delivery, and bioscaffolding [130,131]. The traditional methods to synthesize these NPs are precipitation, hydrothermal, solvothermal, and spray pyrolysis [129]. The precipitation method involves the dissolution of cerium hydroxide in a solution of sodium hydroxide, which results in the formation of precipitates (CeNPs) [132]. In the hydrothermal method, cerium nitrate is hydrolyzed using ammonium hydroxide, and CeNPs are obtained under controlled pH conditions [133]. For example, Magdalane et al. produced CeNPs through the hydrothermal method using cerium nitrate and hydrazine, maintaining the solution pH at 10. The produced NPs showed a cubic fluorite structure with an average particle size of 55–90 nm (SEM measurements) [134]. In the solvothermal synthesis, organic solvents are used inside a chamber under high pressure and temperature to produce NPs of different sizes [135]. Soren and coworkers produced monodispersed CeNPs by a microwave-mediated solvothermal synthesis using 1,4-butanediol as a capping agent and ceric ammonium nitrate as the precursor, yielding NPs with a particle size ranging from 5 to 10 nm (SEM and TEM measurements) [136]. Another way to synthesize CeNPs is by microemulsification method where a polar aqueous medium and a non-polar aqueous medium are mixed in the presence of a surfactant, producing NPs of controlled size and structure [135]. Iqbal et al. synthesized CeNPs via reverse microemulsion synthesis using cerium nitrate, triton X-100 as a surfactant, 2-propanol as the co-surfactant, and cyclohexane as the oil phase. The synthesized NPs had a cubic fluorite structure with an average particle size of 4 nm (Sem and TEM measurements) [137]. Kalaycıoğlu and coworkers used turmeric and different kinds of honey to produce CeNPs by the eco-friendly green synthesis approach. They obtained spherical CeNPs with particle sizes of 1.23, 2.61, and 3.02 nm for the blossom, chestnut, and pine honey, respectively (TEM measurements) [138].

#### 1.1.7. Titanium Dioxide NPs

Titanium dioxide nanoparticles (TiO_2_NPs) are photo-active metallic nanoparticles that are becoming very promising for biomedical applications, including drug delivery systems, cell imaging, genetic engineering, photodynamic therapy for cancer, and as biosensors [139,140,141]. Also, these NPs have a high refractive index, which makes them very attractive for several industries, including coatings, papers, inks, food products, plastics, cosmetics and textiles [142]. There are several strategies to produce TiO_2_ NPs, such as electrophoretic deposition, spray pyrolysis, hydro/solvothermal, sol-gel, and microwave-assisted methods [140]. The electrophoretic deposition involves the movement of charged particles in a suspension medium followed by deposition on a substrate under an applied DC voltage [143,144]. Changing the deposition parameters such as voltage, deposition time, and solvent type can influence the size of the produced particles [144]. Pallo-Sigcha et al. produced TiO_2_ thin film through electrophoretic deposition using aluminum as the anode and boron-doped diamond (BDD) as the cathode with an electrode gap of 1 cm. They produced a film with an average thickness of 8–9 µm [145]. Generally, the spray pyrolysis consists of the passage of the precursor’s flux by a direct flame. It can proceed either by supplemental burners that are mounted near the spray nozzle or by an additional feeding of the nozzle by the oxidant that could be air or pure oxygen and the combustibles [143]. Researchers have produced TiO_2_NPs by flame spray pyrolysis using liquid petroleum gas as fuel. They obtained TiO_2_NPs with a particle size of 0.48, 0.68, and 0.84 µm (SEM measurements) [146]. The hydrothermal method consists in the growth of a single crystal of the desired material [147]. This method is performed in a high-pressure vessel such as an autoclave, being exposed to high temperatures. Shahat et al. produced TiO_2_NPs using the hydrothermal method at low temperatures. The setup consisted of the ultrasonication of commercial titanium dioxide, followed by the use of an autoclave at 75 °C and an oven at 500 °C for 3 h. The obtained TiO_2_NPs were semi-spherical with a particle size ≥ 50 nm (SEM and HR-TEM measurements) [148]. The solvothermal method is very similar to the hydrothermal method, but a non-aqueous solvent is used instead of an aqueous solution of the material. This method allows better shape, distribution, crystallinity, and size control of TiO_2_NPs compared to the hydrothermal method [143,149]. Aguilar et al synthesized TiO_2_NPs via solvothermal method using a thermal oil as the medium and benzylic alcohol as the reagent. TEM images revealed a uniform shape of the NPs with a spherical symmetry with an average particle size ranging from 300 to 450 nm. DLS results demonstrated an average zeta potential ranging from −40 and −80 mV [150]. The sol-gel approach is a wet-chemical method, which can be defined as the conversion of a precursor solution to an inorganic solid through a polymerization reaction induced by water. It is a very promising method to prepare inorganic and organic-inorganic hybrid nanomaterials because it works at low temperatures and allows a homogeneous molecular composition [151]. Also, the NP size and shape are easy to control using this method. Dubey and coworkers synthesized TiO_2_NPs through a sol-gel approach, giving rise to spherical particles with an average size of 13 nm (TEM data) [152]. Alternatively, microwave-assisted methods employ microwaves to generate heat by rotation, friction, and collision of molecules, resulting in an increase of the local temperature [143,153]. Compared to the conventional methods, the microwave heating is an alternative heat source for rapid heating due to its shorter reaction time, higher reaction rate, selectivity, and yield [154]. This radiation can also be applied to the synthesis of TiO_2_NPs through the microwave-assisted hydrolysis of titanium tetrachloride in an acidic aqueous medium [154]. Falk et al. developed TiO_2_ NPs by a microwave-assisted method combining the sol-gel and hydrothermal synthesis. The produced NPs presented sizes ranging from 7 to 28 nm and 13 to 52 nm (particle-size distribution (PSD) measurements) [155].

### 1.2. Silica NPs

Despite all the complexity around the classification of silica NPs, the majority of researchers classify them as inorganic, so in this review that classification will be maintained. Silica has been recognized as safe for use by the FDA due to their known biocompatibility, making them very attractive for pharmaceutical applications [156]. Silica nanoparticles (SiNPs) exhibit excellent properties, including biocompatibility, low toxicity, thermal stability, and scalability, being applied to separate proteins, detect nucleic acids, for drug and gene delivery, and as imaging contrast agents [157,158]. Moreover, these NPs can be applied in other fields such as chemical, biotechnology, environmental remediation, agriculture, and waste water purification [157,158]. There are many different types of SiNPs, such as the conventional non-porous SiNPs, mesoporous silica nanoparticles (MSNs), hollow mesoporous silica nanoparticles (HMSN), and core-shell silica, either with or without surface modification [159].

SiNPs can be synthesized by various approaches, yielding NPs over a size range of 10–500 nm with a variety of shapes and physicochemical properties. The most employed methods are the Stober’s method and the microemulsion method. The Stober’s technique uses a silica precursor, the tetraethylorthosilicate (TEOS), which in the presence of ethanol and ammonium hydroxide undergoes hydrolysis followed by a polycondensation reaction to produce non-porous silica particles with smaller than 200 nm [160]. In addition to TEOS, other low-cost precursors such as sodium silicate solution (SSS) have been used [161]. Gao et al. produced SiNPs via the Stober approach, controlling the obtained NPs particle size by varying the volume of the solvent used, for instance ethanol. SiNPs diameters ranged from 70 to 400 nm (SEM measurements) [162]. Another method for the synthesis of SiNPs is the microemulsion technique, which involves the formation of oil-in-water (O/W) micelles or water-in-oil (W/O) reverse micelles [161]. These micelles stabilized by surfactants such as tweens or pluronics function as nanoreactors for particles synthesis. Therefore, the size of the nanoparticles primarily depends on the volume of these so-called nanoreactors. It is inside these nanoreactors that silica precursors undergo hydrolysis and condensation reactions to form SiNPs. This method allows loading of fluorophores and drugs into the nanoreactors to facilitate drug delivery applications. Koźlecki and coworkers used Tween^TM^ 85 to prepare SiNPs in oil-in-water microemulsion, producing SiNPs with diameters raging between 130 and 500 nm (DLS measurements) [163]. There are other chemical methods that have been employed for the synthesis of SiNPs, including low-temperature vapor-phase hydrolysis [164], spray drying [165], and chemical preparation [166]. The particle size is generally controlled by varying the reaction parameters such as ammonia/sodium hydroxide concentration, mixing speed, or the rate of TEOS addition [158]. Moreover, SiNPs are relatively easy to functionalize, especially due to their high content of silanol groups (Si-OH), which can be easily manipulated as the site of attachment for surface probes [158]. Recently, several biogenic methods have been employed to synthesize SiNPs involving microorganisms and nature-derived substrates [159]. Also, biomass has been investigated to produce SiNPs, like rice straw, husk, and sugarcane bagasse. In this process, silica is initially isolated and transformed into sodium silicate solution [159]. Pieła et al. synthesized SiNPs from corn cobs husks with an efficiency of bioconversion of around 47%, yielding spherical NPs with approximately 40 to 70 nm in size (SEM and STEM measurements) [167].

### 1.3. Organic NPs

Organic NPs are present in nature and are part of many industrial products [168]. They are solid particles composed of organic compounds (mainly lipids or polymeric) with diameter ranging from 10 nm to 1 µm [169]. The research into organic NPs has increased over the years, especially due to the evolution of the pharmaceutical industry [170]. Biopolymer NPs are offering numerous advantages that embrace the simplicity of their preparation from well-understood biodegradable, biocompatible polymers and their high stability in biological fluids during storage [170]. Relevant advantages and limitations of inorganic NPs in the biomedical field are described in Table 1.

#### 1.3.1. *Polymeric micelles*

Polymeric micelles (PM) are by definition polymeric capsules with membranes, considered to be similar to phospholipids due to their hydrophobic bilayer structure [171,172]. They have been applied in the biomedicine field, being very important in delivering highly hydrophobic drugs, e.g., anti-cancer drugs, contrast imaging molecules, and peptides [173]. These NPs have gained popularity especially in drug delivery since their core can be used to solubilize several hydrophobic/hydrophilic compounds, whereas its hydrophilic corona offers protection against drug clearance by inhibiting opsonization. Also, these NPs are used as catalysts and building materials [172]. Basically, micelles are formed due to the monomer structures of the hydrophilic blocks, which join together as well as the non-polar blocks. The hydrophilic blocks in a polar media will turn into the media, whereas the hydrophobic blocks will unite in the center, generating a polymer structure [174]. A micelle with the hydrophilic block on the outside is called a normal phase micelle, whereas a micelle with the hydrophilic block on the inside, for instance in a non-polar media, is called a reversed phase micelle [174]. A great application of polymeric micelles is the low critical micelle concentration (CMC), in which self-assembly takes place to form spherical micelles, showing improved aqueous solubility, sustaining drug release behavior and a decreased cytotoxicity [43,44]. According to the literature, the two most commonly used methods to produce polymeric micelles are the solvent-switch technique and the organic solvent-free technique [172]. In the solvent-switch method polar organic solvents, such as s N,N-dimethylformamide (DMF) and tetrahydrofuran (THF) are used to dissolve the polymers that will produce the NPs. Then, a second solvent, usually water, is added to the solution to promote solution’s hydration. The formation of the polymer vesicles occurs due to the insolubilities of the hydrophobic part of the polymer and the water, leading to the self-assembly of the polymer [172]. Also, the choice of the solvents can influence the size and distribution of polymer vesicles [172]. Wang and coworkers produced polymeric micelles based on the amphiphilic poly(N-2-hydroxypropyl methacrylamide)-block-poly(N-2-benzoyloxypropyl methacrylamide) (p(HPMAm)-b-p(HPMAm-Bz)) via solvent-switch method where DMF, THF, dimethylacetamide (DMAc), dimethyl sulfoxide (DMSO), and acetone were used as the organic solvents. They found that the use of THF and acetone resulted in larger micelles, likely due to their relatively high water–solvent interaction parameters as compared to the other solvents tested. The results obtained showed that the size of all-HPMA polymeric micelles can be easily tailored from 40 to 120 nm by simply varying the formulation properties [175]. Regarding the solvent-free methods, it is important to highlight three approaches, the rehydration, the pH-sensitive polymers, and the polyion complex vesicles (PICsomes). In the rehydration approach, polymeric vesicles can be synthesized using organic solvents like chloroform to dissolve the polymers, followed by a process of hydration with water finishing and evaporation process of the solvent to obtain a thin film, which is very similar to the solvent-switch method. Another way is the bulk swelling where water-soluble polymers are used to prepare polymer vesicles [172]. Du and Armes produced block copolymer vesicles in pure water through the bulk swelling approach using a diblock copolymer, poly(ε-caprolactone)-block-poly [2-(methacryloyloxy)ethyl phosphorylcholine], or PCL-b-PMPC. The resulted vesicles had a hydrodynamic diameter of 40 to 500 nm and an intensity average diameter of 131 nm (DLS results) [172,176].

In the case of the pH-sensitive polymers, polymersomes can be produced by increasing or decreasing the pH value of the solution. This will lead to a shift in the ratio between the hydrophobic section and hydrophilic section of the polymer, causing the polymer to self-assemble into polymersomes [172]. PICsomes are simple to prepare and are formed by the self-assembly of a complex with oppositely charged polyelectrolytes in an aqueous solution. They have been investigated for the future use of polymer micelles as drug carriers. Kishimura produced nano PICsomes by mixing homo-P (Asp-Ap) and polyethylene glycol (PEG)-b-PAsp (fPEG ≈ 8%). The polymeric micelles were a PEG-PIC-PEG three-layer structure with a size range up to 300 nm (DLS and cryogenic phase-contrast TEM results) [172,177].

#### 1.3.2. Chitosan-Based NPs

Chitosan (CS) is a marine-derived cationic polysaccharide, approved by the FDA for wound dressing applications and cartilage repairing formulations [178]. CSNPs are considered a potential and effective tool for drug delivery, due to their biocompatibility, biodegradability, low toxicity, versatility, and ease of processing [178,179]. Furthermore, CSNPs are also used in the food industry, marine biofouling, paint industry, agriculture, water treatment, as well as in the textile industry [180]. Ionic gelation is the most popular procedure to produce CSNPs. It is a self-assembly process driven by electrostatic interactions between aqueous solutions of charged molecules such as CS (polymeric molecule with charged or chargeable groups) and small molecules (e.g., tripolyphosphate (TPP)) carrying opposite electrical charges [178,181,182]. It is an easy, low-cost, and versatile approach that requires a simple and easily scaled-up apparatus, enabling multiple compounds incorporation with high stability, efficiency, and controlled release [178,183]. Essa et al. developed wasp CSNPs via ionic gelation with an average hydrodynamic diameter of 477 nm (DLS results) and a zeta potential of 43.9 mV. The TEM results revealed that the NP’s size was lower than the estimated by DLS being around 200–280 nm [184]. Emulsification is another possible method used to synthesize CSNPs that implies the mixing of one liquid phase into another totally or partially immiscible by resorting to surfactants, which reduce the interfacial tension between the two liquid phases to reach stability [178,185,186]. Furthermore, the non-aqueous phase is removed by evaporation under low pressure, vacuum, or solvent extraction using a large volume of water, leading to the formation of NPs dispersed in the water phase. Therefore, formed NPs are collected, washed, and freeze-dried for storage [178]. In some cases, hybrid methods like emulsification followed by ionic gelation can be applied to stabilize the hydrophilic particle surface [178,185,186,187,188]. Trombino et al. developed CSNPs by membrane emulsification with spherical shape and sizes of 1.9 µm (DLS measurements) [189].

#### 1.3.3. Liposomes

Liposomes are spherical particles composed of one or more lipid and/or bilayers, containing spacing between the bilayers [170]. A liposome comprises a tiny vesicular structure that closely resembles the structure of a cell membrane. They are usually made of phospholipids, consisting of two tails and a head region. The head represents the hydrophilic part of the molecule, whereas the tails represent the hydrophobic fatty acid portion of the molecule. Liposomes are very dynamic structures and fluid entities that result from highly specific supramolecular assemblies, being used on a large scale in drug and gene delivery, as well as for several analytical and diagnostic purposes [173]. Their structure can contain lipophilic as well as hydrophobic and amphiphilic molecules, which can be very useful in incorporating compounds with different solubilities in the spacing together [190]. The molecules can be transported to the site of action where the bilayer fuses with the other bilayer such as a cell membrane. These NPs can be classified according to their size and their lamellarity (number of bilayers): the ones containing only a single bilayer membrane are called small (>30 nm), while large uni-lamellar vesicles are in the range of 30–100 nm [170]. Also, their properties differ with lipid composition, surface charge, size, and method of preparation. Moreover, the choice of the bilayer components determines the rigidity or fluidity and the charge of the bilayer; for example, unsaturated phosphatidylcholine species from natural sources (egg or soybean phosphatidylcholine) give rise to much more permeable and unstable bilayers, whereas the saturated phospholipids with long acyl chains (for example, dipalmitoylphos phatidylcholine) form a rigid, rather impermeable bilayer structure [191]. Liposomes are extensively used as carriers for a vast variety of molecules in cosmetic and pharmaceutical industries. Additionally, food and farming industries have also studied the use of liposomes for the encapsulation of unstable compounds, including antimicrobials, antioxidants, flavors, and bioactive elements, and shield their functionality [192,193]. Due to their biocompatibility, biodegradability, low toxicity, and aptitude to trap both hydrophilic and lipophilic drugs, liposomes have gained much interest as a drug delivery system [194]. Regarding the liposome preparation techniques, the conventional methods involve several steps, including: dissolution of lipids in an organic solvent, drying-down of the resultant lipidic solution from the organic solvent, hydrating the lipid with an aqueous media, downsizing and/or change in lamellarity, post-formation processing (sterilization, purification), and finally the characterization of the final nanoformulation product [195]. The current tendencies in liposome production include the freeze-drying method, supercritical fluid-assisted method, microfluidic method, and the membrane contactor method. In the freeze-drying method, the aqueous solution containing the liposome formulation is frozen, followed by removal of ice by sublimation allowing the preservation of the shelf stability of the liposome [195]. Guimarães and coworkers developed liposomes encapsulating anticancer drugs, namely methotrexate (MTX) and doxorubicin (DOX) in the aqueous core and tamoxifen (TAM) in the lipid bilayer. Sucrose proved to be adequate for the cryo/lyoprotectant function of these liposomes. In this work, the liposomal suspensions were stored for 6 h at −80 °C in a Corning® CoolCell™ to achieve a slow rate of freezing of ≈−1 °C/min. The freeze-drying process was performed for 24 h at −50 °C in a chamber with 6 Pa. After the freeze-drying process, liposomes with sucrose encapsulating drugs revealed high physical stability, maintaining their narrow and monodisperse properties [196]. In the supercritical fluid method, the dissolution of the lipids in supercritical carbon dioxide is performed under high pressure, in general 250 bar. Then, the obtained supercritical homogeneous solution is successively expanded at 60 °C, with the addition of ethanol. Lastly, the expanded liquid is mixed with a water phase and liposomes are formed [195]. Penoy et al. developed a supercritical fluid technology for liposomes production involving carbon dioxide as a dispersing agent. A quality by design strategy was employed to find the optimal production conditions and two conditions were considered optimal: Lipid concentration around 5 mM, 10 mL of dispersion, temperature of 80 °C and 156 bar of carbon dioxide pressure and 45 mM of lipid concentration, 14 mL of dispersion, 80 °C and 240 bar of carbon dioxide pressure. With the first condition, liposomes having sizes near to 200 nm and polydispersity index (PdI) < 0.36 were produced, while with the second approach the formed liposomes were similar but, taking into consideration an industrial point of view, enabled the production of more concentrated batches with higher encapsulation efficiency [197]. Another alternative is the microfluidic method, where lipids are dissolved in low toxicity solvents such as ethanol or isopropanol and then propelled within microscopic channels. The liposomal solutions are placed between two aqueous streams in a microfluidic channel, generating a laminar flow and a mixing at the two liquid interfaces leading to the self-assembly of lipids into vesicles, called liposomes. This method allows a precise control of mixing and fluid flow rates, producing in general small monodisperse liposomes with controllable sizes and distributions [195]. Xu and coworkers fabricated curcumin loaded liposomes through a microfluidic approach using newly designed microfluidic swirl mixers able to prepare liposomes at a larger scale (up to 320 mL/min or 20 L/h). The size of the produced liposomes ranged between 50 and 134 nm (Cryo-TEM results) [198]. Lastly, the membrane contactor method consists of two pressurized vessels, one for an organic phase containing lipids, and the other for an aqueous phase separated by a special porous glass membrane, having pore sizes that allow the flow of the organic phase [195]. Laouini et al. used a polypropylene hollow fiber module as a membrane to produce liposomes encapsulated with a hydrophobic drug model—spironolactone. TEM results indicated the formation of spherical oligolamellar vesicles with an average size of 113 nm and a zeta potential of −43 mV (DLS and electrophoretic light scattering (ELS) results) [199].

#### 1.3.4. Dendrimers

Dendrimers are synthetic polymers, hyper-branched, and predominantly three-dimensional macromolecules [200]. They have a central core that consists of repeating units and many terminal functional groups that are located in the outer region of the macromolecular architecture, normally with 1–2 nm in size [173]. In comparison with other types of NPs, such as micelles and liposomes, these structures do not have a fully hollow core, but are from the inside-out expanding polymeric networks of repeating units. The way the structure is built is often compared to an onion, where the shells represent a repeating unit attached to the next inner cell, becoming smaller at the center. The total structure can be further divided into three main structural components, the core, the outer shell, and the multivalent surface. The core is in higher generation dendrimers protected from the surroundings generating a dendrimer-specific micro-environment. The outer shell is located just below the surface and has its own well-defined micro-environment. The multivalent surface is characterized by the large number of potential active sites [201,202,203]. Dendrimers can be synthesized mainly by two methods: divergent and convergent methods. The divergent methods comprise the growth of several branches initiating in a radial manner from the core to the peripheral region. On the other hand, in the convergent methods, the direction of growth is completely the opposite to the divergent method, which is extending from the peripheral regions to the core. The optimization of size, shape, branching density, and surface functionality distinguishes them as ideal carriers in drug delivery [202,203]. Dendrimers are widely used within the pharmaceutical and medical areas. The applications include contrast agents, such as for magnetic resonance imaging (MRI), but it is more frequently employed as a drug delivery system. Also, they have been used in catalysis reactions, sensing, photonics, and molecular electronics [204]. The future of dendrimers synthesis goes through the implementation of click chemistry, which in the last years has received a lot of attention as it offers new efficient approaches as well as the possibility for scalability [205,206].

Incorporating NPs (made by several materials) within polymer-based fibers enables the production of functional fibers that present the inherent benefits of both NPs and fibers. It has been reported that these hybrid scaffolds composed by NPs and fibers enhance their performance, cellular interactions, and biological properties compared to similar composites without incorporated NPs. So, these scaffolds have rapidly emerged in tissue engineering, regenerative medicine, and wound healing [4,207,208]. In the next section, natural and manufactured fibers will be described as well as the different methods of fiber formation.

## 2. Fibers

A fiber is defined as units of matter characterized by fineness and flexibility with a high ratio of length to thickness [209,210]. But it is more often referred to as the basic unit of matter, either natural or manufactured, that forms the basic element of fabrics and other textile structures [211]. In technical terms, a fiber is defined as being an elongated structure with a length that exceeds its breadth. According to their origin, fibrous materials can be classified in two main groups: natural and manufactured [211]. Natural fibers are fibers that exist as such in the natural state, and can be divided into animal, plant, and mineral-based fibers. On the other hand, manufactured fibers are made by processing natural or synthetic organic polymers into a fiber-forming compost being classified as synthetic, natural, or others, which include carbon, glass, metal, and ceramic fibers [211,212].

Fibrous structures have been increasingly adopted in biomedical applications such as drug delivery, artificial implants, tissue engineering, cancer treatment, and rehabilitation of the human body [209].

### 2.1. Natural Fibers

Natural fibers are produced by plants, animals, and geological processes. They are biodegradable over time and are classified according to their origin into animal-based, vegetable-based, and mineral fibers. An interesting fact is that over half of the fibers produced annually are natural fibers, mainly cotton [213]. Natural fibers have been widely used as reinforcement biocomposites in drug delivery, tissue engineering, and organ implants due to their biocompatibility, non-toxic nature, and good mechanical properties [214].

The use of natural animal fibers in textile materials began very early in history. Animal fibers are made from silk, wool, angora, mohair, and alpaca. Natural fibers, the source of which is the pelage of animals, exhibit a variety of morphological features that may be used to identify the particular family the hair originated from [210,211]. Animal fibers consist of animal hairs and fibers from animals’ secretions. Their main chemical components are proteins that are formed by a series of amino acids through polymerization of peptide bonds into macromolecular chains; thus, animal fibers are also called natural protein fibers. The animal fibers are mainly classified as hair fibers and silk fibers. Depending on the application, animal fibers can be considered good raw textile materials due to their high elastic properties, warmth retention, water absorption, and soft luster. They can also be interlaced into many types of high level of yarns and garments throughout four seasons of a year and surely other decorative and technical textiles [215]. Animal-based fibers have been explored mainly for tissue engineering applications, namely of bone, neural, vascular, skin, cartilage, ligaments, tendons, ocular, bladder, and cardiac tissues [212,216]. Moreover, the use of natural fibers mixed with biodegradable polymers can produce joints and bone fixtures [213].

Plant fibers are found as structural elements in all higher plants and are composed of cellulose, hemicellulose, lignin, and waxes in their structure [214,217]. Plant fibers are classified according to their source in plants and include cotton, hemp, jute, flax, ramie, sisal, and bagasse [214]. The structural elements of higher plants are formed by elongated fiber cells, which give strength and shape to the tissues of stems, branches, and roots, but also to leaves, flowers, or fruits (soft tissues). Furthermore, plant fibers owe their mechanical strength to the special design of the cell architecture and the cellulose fibrils contained in cell walls. Cell walls are composed of polysaccharides, glycoproteins, and phenolic compounds forming in the fiber’s thick layers of structural material around the protoplast [217]. Plant fibers are renewable and biodegradable being very important for sustainable products. Moreover, these fibers have been frequently used in the fabrication of biocomposites, being endowed with high strength to weight ratio, non-corrosive nature, high toughness, renewability, and sustainability. These biocomposites are already being used for biomedical applications, such as drug/gene delivery, tissue engineering, orthopedics, and cosmetic orthodontics, because they have the potential to regenerate traumatized or degenerated tissue or even entire organs [218].

Mineral fibers are other naturally occurring fibers, but they also constitute a slightly modified fiber procured from minerals. They can be divided into three main categories: asbestos, which is the only naturally occurring mineral fiber-like serpentine, amphiboles, and anthophyllite. These types of fibers have been used as composite reinforcement of other fibers, namely in bone tissue engineering, dental prosthesis, and tooth restoration [219,220].

### 2.2. Manufactured Fibers

Manufactured fibers are the fibers produced from organic and inorganic raw materials. These fibers are produced by extruding a fiber-forming substance, through a hole or holes in a shower head-like device called a spinneret, and this process is called spinning. The organic materials can be natural or synthetic polymers, while the inorganic compounds include glass, metal, basalt, quartz, and other composites. They are manufactured in the form of monofilaments, staple fibers, and filament yarns [214,221].

In general, they are more durable than most natural fibers, and their properties can be easily tailored to the intended application by varying the chemical composition and the processing conditions. Also, compared to natural fibers, manufactured fibers are usually more water, stain, heat, and chemically resistant. Natural fibers are much more sensitive to chemical degradation than manufactured fibers because they are biodegradable, can be attacked by several bacteria and fungi, and break and wear down over time [209]. These fibers and associated properties are widely applied in wound healing, personal protective equipment, tissue engineering, drug delivery, scaffolds, and antimicrobial materials, among others [2,220,222,223].

#### 2.2.1. Natural Polymers as Building Blocks for Manufactured Fibers

In fibers manufactured from natural polymers, their production process can greatly influence the resulting fibers, including their physical, chemical, and mechanical properties [214]. As an example, cellulose can be manufactured into cellulose acetate (CA), lyocell, among others, depending on the process used to fabricate the fibers [214]. Several natural polymers can be used to produce such fibers, from which cellulose, CA, CS, collagen, gelatin (GN), and alginate will be reviewed in this section.

Cellulose is the most abundant polymer found in nature [224,225]. It is biosynthesized by several organisms, such as lower plants, sea animals, bacteria, and fungi [225]. This polymer consists of β-D-glucopyranose (glucose) units linked together by β-(1,4) glycosidic bounds. It is characterized by thermal and chemical stability, good mechanical properties, high biocompatibility, non-toxicity, biodegradability, high hydrophilicity, and chemical versatility [226,227,228]. Cellulose can be used for several biomedical applications, such as coatings, wound dressings, tissue engineering scaffolds, among others. CA is an acetate ester derivative of cellulose, and is one of the most important cellulose derivatives with a wide range of biomedical uses, such as wound dressings, antimicrobial membranes, biomedical nanocomposites, among others [229]. It is characterized by biodegradability, biocompatibility, good mechanical properties, non-toxicity, high affinity to other polymers and/or cells, good hydrolytic stability, relatively low cost, and excellent chemical resistance [229].

CS has been explored in recent years for several applications, including biomedical. It can be obtained through a process of deacetylation of chitin, in a reaction with high temperatures and alkaline media conditions [178]. The skeleton of CS is formed by glucosamine and N-acetylglucosamine connected by a 1,4-glycosidic bond, leading to a linear polymeric structure [178]. This natural polymer is FDA-approved for two applications: nutritional use and wound treatment [230]. It possesses very interesting properties, such as biocompatibility, biodegradability, antimicrobial activity, analgesic, regenerative, antioxidant, and hemostatic, making CS a valuable option for wound dressings [178,231,232,233,234]. Moreover, its cationic attributes have made CS an excellent choice for cancer therapy and drug delivery [230].

Collagens are the most abundant extracellular matrix proteins in mammals, representing about 30% of total protein mass [235,236]. Collagen is a complex supramolecular structure, occurring in diverse morphologies across different tissues, conferring them a wide range of biological functions. The collagen molecule is composed of a triple helical region and two nonhelical regions at either end of the helix. Also, evolutionary branching has led to multiple genetically distinct collagen types [236]. Moreover, FDA approved a collagen-based implant for bone and a bioengineered skin [237]. This natural polymer is endowed with very valuable features including biodegradability, biocompatibility, adequate mechanical properties, and cell activity, making it an excellent choice to apply in wound healing, tissue engineering, surface coating, and skin supplementation [237].

Another very promising natural polymer for biomedical applications is GN. It is a natural polymer, FDA-approved as a pharmaceutical excipient and food ingredient, and water-soluble, frequently used in biomedicine being also endowed with biocompatibility, biodegradability, low antigenicity, and cost effectiveness [238,239]. GN is extracted from porcine, bovine, or fish collagen (mainly type 1 collagen) Collagen is hydrolyzed to protein fragments by acidic or basic treatment, producing type A or type B gelatin, respectively [240]. Moreover, this polymer contains bioactive sequences derived from collagen (e.g., Arginylglycylaspartic acid (RGD) peptides and matrix metalloproteinase (MMP)-sensitive degradation sites) and several functional groups (e.g., primary amine, carboxyl and hydroxyl groups) that enables its modification with therapeutic agents, increasing its applicability as a versatile material for wound healing and regeneration [238,241,242,243]. Also, GN is frequently applied in the production of hydrogel-like films for controlled drug release [239,244,245].

At last, alginate, which is an FDA-approved polysaccharide used in food, medicine, and pharmaceutical applications [246]. It is a naturally occurring polymer that can be obtained from kelp or *Sargassum algae* of brown algae and some bacterial strains, being composed by β-D-mannuronic acid and its C5 epimer α-L- guluronic acid linked together by a 1,4- glycosidic bond [247]. Alginate has excellent biodegradability, biocompatibility, non-toxicity, and the capability of gelling has been widely used as a biomaterial for biomedicine purposes, including wound healing, tissue regeneration, drug delivery, and 3D bioprinting [247].

#### 2.2.2. Synthetic Polymers as Building Blocks for Manufactured Fibers

Synthetic fibers are synthesized from chemical compounds, with their development being dependent on the advances in polymer synthesis, spinning methods, and appropriate solvents. Among the numerous classes of synthetic fibers, this review will point out the most relevant synthetic polymers used to build manufacture fibers, such as polyamides, polyesters, polyacrylonitrile (PAN), polyaniline (PANI), polycaprolactone (PCL), polyethylene glycol (PEG) and poly(butylene terephthalate (PEOT/PBT), polyethylene oxide (PEO), poly (lactic acid) (PLA), poly (lactic-co-glycolic) acid (PLGA), poly (l-lactic acid) (PLLA), polypropylene (PP), polyurethane (PU), polyvinyl alcohol (PVA), and poly (vinylpyrrolidone) (PVP).

Polyamides can occur both naturally in wool and silk or synthetically [248]. Synthetic polyamides are very important polymers for several applications including biomedical. They are constituted by monomers binding to amide groups and are usually fabricated via a process of condensation polymerization [248,249]. Such fibers have several interesting characteristics like biocompatibility, adequate chemical stability, mechanical strength, flexibility, toughness, and resistance that makes them an excellent option for sutures, catheters, wound healing, drug delivery, and most recently tissue engineering [248,249]. Moreover, fabrics made of polyamides are widely used to produce protective clothing, including for heat and flame protection and medical protective equipment [5,248,250,251,252].

Polyesters are also synthetic polymers that can be obtained by condensation reactions, having a characteristic ester linkage in its backbone structure [253]. These polymers have gained significant attention in the medical field, due to their attractive features, such as biodegradation, biocompatibility, and ease to modify and to synthesize. They can be found in dental implants, soft tissue sutures and staples, tendon and ligament reconstruction, bioimaging, and protective clothing [5,250,254].

PAN is a liner synthetic polymer with thermoplastic properties, optimum solvent, and chemical resistance as well as good mechanical properties [214,255]. This polymer is produced by polymerization of acrylonitrile as a monomer and main component [214]. It is a great versatile polymer due to it high carbon content that guarantees its high biostability and resistance to degradation [256]. PAN-based materials are mainly used in implants, limbs, components, and replacement filler materials due to their unique properties, such as high strength, light weight, stiffness, and resistance to fatigue [256].

PANI is a synthetic polymer derived from the polymerization of aniline, known for its simplicity, stability, and ability to be doped by protonic acids [257]. According to its oxidation state, this polymer can form five individual structures: leucoemeraldine, protoemeradine, emeraldine, nigraniline, and pernigraniline [258,259]. As such, it is fit for various applications, including electromagnetic shielding, photothermal therapy, chemical sensor, anticorrosion coating, and microwave absorption [260,261,262]. PANI is also used to produce a great variety of products, including fibers for textiles or hollow fibers for reverse osmosis [214]. More recently, PANI has been used for electrical conductivity circuits. It also presents low toxicity and biocompatibility, antioxidant, antimicrobial, and antiviral abilities, making it ideal for drug delivery, cancer therapy, and tissue engineering [263].

PCL is a linear synthetic biodegradable aliphatic polyester with many applications in prosthetics, sutures, and drug delivery systems. It is an FDA-approved polymer for clinical use in humans, endowed with excellent biodegradability, compatibility with a wide range of other polymers, good processability, which enables fabrication of a great variety of structures, and is cost-effective [264]. PCL is suitable for controlled drug delivery systems given its high permeability to many drugs, excellent biocompatibility, and its ability to be fully excreted from the body once bioresorbed [264]. It possesses an excellent thermal stability and is susceptible to surface modifications [265]. This polymer has a semicrystalline regular structure, which increases its toughness, and with its amorphous domains being in the rubbery state [266].

PEOT/PBT are multi-block copolymers with thermoplastic elastomeric properties, obtained by phase separation of the hydrophilic and hydrophobic segments in the polymers, and by variation of the copolymer composition, the physical properties of PEOT/PBT can be tuned in a wide range. They exhibit excellent thermal and mechanical properties, being widely used as biomaterials. These copolymers are good candidates as scaffolds for tissue engineering since they induce a weak inflammatory response and have a slow degradation profile under in vivo conditions [267,268].

PEO, also known as PEG, is an FDA-approved polymer for clinical use, since it is non-toxic and non-immunogenic [269]. It is a bioadhesive and non-ionic hydrophilic polymer, presenting fast hydrating hydrophilic properties, being widely used for surface modification of biomaterials and induction of cell membrane fusion. Several PEO-based copolymers have been used for drug delivery applications [270]. Moreover, PEO presents good water solubility, safety, high swelling capacities, and a thermoplastic behavior [271,272]. Also, PEO forms a viscous gel upon hydration and its gel-forming properties endow PEO with resistance to intravenous abuse, limiting the loading of the gel into a syringe [273].

PLA is another FDA-approved polymer for clinical use, due to its biocompatibility, biodegradability, and aliphatic behavior, being produced from renewable resources. PLA can exhibit three stereochemical forms: poly(l-lactide) (PLLA), poly(d-lactide) (PLDA), and poly(dl-lactide) (PDLLA) [274,275]. This polymer also presents transparency, flame-retardant, and oil- and water-resistant properties. PLA is extensively used in biomedical applications, replacing conventional petrochemical-based polymers in industry [276]. Since PLA is melt-spinnable, stress crystallizes with drawing, its largest application consists of fiber and film manufacturing [277,278]. In addition, PLA’s elastic modulus is very similar to human bone, being an ideal matrix for bone scaffolds, temporary and long-term implants, along with bone screws, anchors, prostheses, vascular grafts, and drug encapsulation and delivery [277,278]. PLGA results in a combination polyester of PLA and poly (glycolic acid) (PGA). PLGA is available with an ester or acid end group, being resistant to hydrophilic cleavage. The ratio of PLA:PGA influences PLGA properties. For instance, higher proportions of PLA increase the degradation rate of PLGA, whereas higher PGA proportions make the polymer easily degradable. PLGA is greatly amorphous, presenting a glass transition temperature of 50 °C as well as a good solubility in organic solvents, such as acetone, ethyl acetate, and dichloromethane [279,280]. PLGA is commonly applied in biomedical applications because it is easily broken into PLA and PGA, presenting biocompatibility and minimal toxicity. Furthermore, it has been applied as different formulations, including membranes, sponges, and gels. Several reports in the literature address good results of PLGA when applied as orthopedic implants, accelerating bone formation and articular healing in rat models [280,281]. PLLA is a biocompatible and biodegradable synthetic polymer that has gained considerable attention since it is eco-friendly and a promising alternative to other thermoplastic polymers, including polyethylene (PE), polypropylene (PP), and polystyrene (PS) [282]. PLLA presents good mechanical properties, making it suitable for a wide range of applications. Nevertheless, its high elastic modulus limits plastic deformation. The wettability of this polymer must also be taken into consideration when selecting for tissue engineering applications, since its high hydrophobicity can interfere in cell adhesion, resulting in lower interactions between body fluid and the biomaterial [283].

PP is a stereoregular, thermoplastic synthetic polymer with a low melting point, commonly applied in many industries, being considered the fourth largest volume artificial fiber. PP is mainly used as carpets, geotextiles, ropes, and reinforcement fibers [284]. This polymer can be isotactic, syndiotactic, or atactic, presenting helical chain formations. In addition, it presents a melting point range of 160 °C to 170 °C and displays excellent resistance to chemicals and low moisture absorption. PP has also been extensively used in the biomedical field, as sutures and meshes applied in urogynecology and hernia repair, strengthening weakened tissues [284,285].

PU is composed of a chain of organic units joined by carbamate (urethane) links. It is formed by combining two bi-functional monomers, one containing two or more isocyanate functional groups, and other containing two or more hydroxyl groups. This polymer is widely used to produce textile fibers and foam materials [214]. In the medical field, due to its high biocompatibility, blood compatibility, and duration, PU has been an excellent candidate for the preparation of drug delivery systems and biomedical devices such as catheters, heart valves, vascular prostheses, among others [286].

PVA is a non-toxic, semi-crystalline, biocompatible, and biodegradable synthetic polymer. PVA is obtained by hydrolysis of polyvinyl acetate, which properties are influenced by polymerization and hydrolysis conditions [287]. This polymer has several applications, including paper coating, textile sizing, dialysis membrane, wound dressing, and artificial skin, due to its high oxygen and aroma barrier properties, high tensile strength and flexibility, excellent film forming, and adhesive properties [288].

PVP is a synthetic polymer obtained by polymerization of n-vinylpyrrolidone [289]. PVP is also a non-toxic, non-ionic, inert, temperature-resistant, pH-stable, and biocompatible polymer, showing a complex affinity for hydrophilic and hydrophobic drugs [289,290]. Such a polymer has gained much attention towards its use in pharmaceutical, biomedical, cosmetics, and food industry [291]. Different PVP-based drug delivery systems have been used for oral, topical, transdermal, and ocular administration. PVP is also applied in delivery of genes and can be coupled with metal particles for regenerative medicine and targeted delivery [292].

### 2.3. Fiber Formation

Spinning techniques consist of the use of a spinneret in which extrusion occurs, forming continuous filaments [293]. Such techniques involve principles of engineering and material sciences and have been continuously evolving during recent past years [294,295]. Four of the most common spinning techniques are discussed in the next sections.

Electrospinning allows the production of fibrous mats with large surface areas to volume ratios, controlled porosity and pore sizes, along with controlled morphologies and chemical/mechanical properties, making it a very promising technique for biomedical purposes [296]. This fiber producing method uses electrostatic force to stretch fibers from a polymer solution. In general, the electrospinning setup consists of three main components, a high voltage power supply, spinneret, and a fiber collector [297]. The high potential (kV) is applied between the spinneret and the collector. Here, the positive electrode of the power supply is attached to the needle to charge the polymer solution during extrusion, while the other is connected to the reverse polarity collector, and these parts are separated at an optimum distance [21]. When the applied electrical field overcomes the surface tension of the droplet, a charged jet of the polymer solution can be expelled from the tip of the needle. The jet grows longer and thinner, with an extended high-diameter loop, resulting in polymer solidification due to solvent evaporation. Finally, as the jet reaches the collector, fibers solidify [298].

Dry-spinning starts on the dissolution of a polymer in an organic solvent, such as ether or acetone. Then, the solution is blended with additives and filtered, resulting in a viscous polymeric solution, named the “dope” solution. The dope solution is followed by filtration, de-aired, subjected to pre-heated processes, and finally pumped by filters, achieving a specific consistency, and extruded in a spinning tube [299,300]. During the extrusion process, fiber-forming substances go through fine orifices of a metallic plate, called a spinneret, at controlled rates. Jets of the polymeric solution contact with a stream of hot gas, and the solvent quickly vaporizes, whereas polymer concentration in the solution is increased and it is solidified without further drying [301]. While the viscous filament streams enter gas flow, solvent evaporates from the surface, forming a solid skin and with further evaporation during downward passage through gas flow, solidified fibers result in a bone shape of dry-spun fibers. Finally, solidified fibrous filaments are drawn-off by rotating rolls and put onto bobbins, with simultaneous stretching [300,301,302]. Dry spinning is more suitable for polymers vulnerable to thermal degradation and unable to form viscous melts. Acetate and triacetate fibers, in addition to aramid and spandex fibers have been successfully produced by dry-spinning [295,301]. The process is considered more complex, in comparison with other spinning methods, due to mass transfer mechanisms in solvent evaporation and filament formation [294].

Melt-spinning is considered an economic process, due to its simplicity and absence of solvents. Polymer pellets are fed into an extruder, containing a screw for melting using heat, and the polymer melt is pumped through a spinneret by means of pressure. The polymer is then contented with cold air and the melted mass is solidified into fibers. Extruded filaments are usually followed by mechanical drawing, resulting in alignment of molecular orientations and improving physical and mechanical properties of the filaments [303,304]. Poly (ethylene terephthalate), polyurethanes, polyolefines, and polyamides fibers have been successfully melt-spun. Nevertheless, this technique presents limitations when applied in the production of biostructures, including poor control over specific temperatures of melt during the spinning process, along with thermo-mechanical history of melt and final fiber morphology [295,305].

Finally, wet-spinning is based on the non-solvent-induced phase inversion method, during which a polymeric solution is injected through a spinneret and extruded into a coagulation bath of a non-solvent or poor solvent of the polymeric solution. As a result, the solution quickly solidifies and precipitates, forming fibers with a wide range of diameters. Also, this technique is capable of generating hybrid structures with different levels of organization and particular arrays of chemical and physical properties [306,307,308]. Several natural polymers, including alginate, cellulose, and gelatin, have been successfully wet-spun for biomedical applications. With wet-spinning, fibers are produced with large diameters and structures with tunable porosity [295,309]. Furthermore, difficulties related to the establishment of optimal processing parameters and polymer thermal degradation can be circumvented, on the contrary with melt-spinning and electrospinning, respectively [310,311,312,313].

Fibers and textiles have been widely investigated for pharmaceutical/medical purposes, including drug delivery systems, gene delivery systems, wound dressings, implantable devices, bone and cartilage substitutes, sensors, among others [295,314]. Much of this growth is due to nanotechnology enabling the preparation of fiber-forming polymers to produce nanofibers and/or the incorporation of nanoparticulate agents into fiber and nanofibers [178,295,314].

## 3. NPs Integration into Fibers for Their Intended Biological Effects

Taking into consideration the aforementioned content of NPs and fibers’ classification and production methods, the integration of NPs into fiber-based systems gathers huge potential for applications in biomedicine. In the following sections, examples of biological effects of fibers functionalized with NPs are provided (Table 2).

### 3.1. Microbial Balance

In recent years, much investigation has been driven towards the detailed study of the human microbiota, which consists of the microbial communities that inhabit our body and are vital to maintain homeostasis [315]. It has been demonstrated that an imbalance often called dysbiosis in the composition of host-associated microbiota is connected to several human illnesses, including in the skin and vagina, among others. Although the definition of a healthy microbiome, and by consequence an unhealthy one, is not yet well understood, the concept of dysbiosis can be defined as a compositional and functional alteration in the humans’ body microbiota with associated disease compared to healthy individuals [316]. Dysbiosis often provokes a loss of beneficial microorganisms, an expansion of pathogens, and a reduced microbial diversity, which can lead to inflammatory states and pathologies [315,317]. Studies have revealed that skin commensals are key microorganisms to maintain the epithelial barrier function, regulate the host immune system, and to offer protection to invading microorganisms. So, the microbial composition of skin wounds clearly affects the process of wound healing, and a balance between different types of organisms is essential to promote skin health and regeneration [318]. Regarding wounds, the most studied are chronic wounds, which are typically colonized by polymicrobial biofilms that encourage pathogenic microbial growth and disrupt the wound healing process [319]. With that being said, it is very important to find therapeutic strategies to combat the growth of those pathogens, helping the skin repair and regeneration, and improving the coordinated events of wound healing. Most of these strategies rely on the use of wound dressings that provide a temporary protective physical barrier, give moisture to optimize re-epithelization, and absorb wound exudates. Also, the current wound dressings offer additional benefits to the patients such as pain relief and antimicrobial properties [320]. The incorporation of a fibrous structure in a wound dressing has gained popularity since they do not only provide physical protection to the wound but also have the ability to be combined with different types of drugs and nanoparticles [321]. Also, the release profile can be controlled and adjusted by modifying the types and compositions of the materials that constitute the fibers [321]. Nanoparticles present various novel approaches for regenerative medicine and are being sought for their biocompatibility, antimicrobial properties, targeted drug delivery, and non-toxicity [322]. In a study conducted by Wang et al., spun PCL/gelatin nanofibrous membranes were produced trough electrospinning, yielding nanofibers of ≈ 560 nm in diameter. Then, mercaptophenylboronic acid-activated AuNPs (MBA-AuNPs) (via one-pot synthesis method under the mechanism of reduction of HAuCl_4_ by NaBH_4_ in methanol, with ≈1.8 nm of diameter) were doped onto the surface of the previously prepared nanofibers for designing multidrug-resistant wound dressing. In vitro testing revealed antibacterial efficiency against Gram-positive bacteria (growth inhibition zones after 24 h of contact with Staphylococcus aureus and MDR *S. aureus*), biosafety (no toxic effects on HUVECs and NIH 3T3 cells and no hemolysis in rat blood), further allowing the survival and proliferation of human endothelial cells. In vitro studies showed 89% and 98% of BALB/c mice wound closure in 14 days, with gauze and with PCL/gelatin nanofibers functionalized with MBA-Au NPs, both with S. aureus and multidrug-resistant (MDR) S. aureus infection. Bacterial growth inhibition was clearly perceived, enabling a faster wound remodeling rate with appearance of hair follicle and sebaceous glands in the wound tissues [222]. PVA/CS nanofibers were fabricated by electrospinning with ≈327 nm in diameter (SEM results). Then, carboxymethyl CSNPs (prepared by electrostatic droplet, with d ≈ 164.6 nm obtained by TEM measurements) were blended in the PVA/CS solution prior to electrospinning. Also, an antibacterial peptide, OH-CATH30 was loaded into the NPs. In fact, the loaded nanofibers exhibited an appropriate degree of swelling for wound healing purposes. In vitro studies revealed a cumulative release of the OH-CATH30 around 66% in 24 h and antibacterial efficiency (inhibition rate of 80% for *E. coli* and *S. aureus*) and no cytotoxicity effects towards human epidermal keratinocytes (HaCaT cells). In vivo studies showed around 98% of KM mice wound closure in 12 days, and the histopathological analysis confirmed that was an acceleration of the re-epithelization and collagen deposition, which promoted wound healing [323]. Another example resorts to the fabrication of core-shell poly (L-lactide-co-caprolactone) (PLCL) nanofibers encapsulating ZnONPs and oregano essential oil as a multifunctional membrane to promote diabetic wound healing. PLCL nanofibrous membranes were produced via electrospinning, yielding nanofibers of ≈1.04 µm in diameter. ZnONPs (purchased with ≤40 nm in size) and oregano essential oil were blended with the polymer solutions prior to core-shell electrospinning. These exhibit adequate tensile strength and wettability for use as wound dressing. In vitro testing showed adequate release rate of Zn^+2^ (621.2 µg in 6 h, 311.8 µg in 66 h), antioxidant potency, antibacterial efficacy (99% and 98% growth inhibition of *E. coli* and *S. aureus*, respectively), also allowing the survival and proliferation of 3T3 fibroblast cells. In vivo studies revealed 89.7% diabetic rats wound closure in 15 days without bacterial infections. These bioactive membranes showed strong antibacterial potential and successfully closed the wound with complete epithelization, granulation tissue formation, neo-vascularization, and collagen deposition [324]. Another case where a microbial balance is crucial to prevent pathologies is in the female vagina. It is well known that the vaginal microbiome is essential to maintain a normal physiological environment for the woman and indispensable for a successful reproductive process [325]. The vaginal microbiome is a dynamic microecosystem that is in constant fluctuation due to many factors, including the menstrual cycle, gestational status, use of contraceptives, and sexual activity [325,326]. Several Lactobacillus species live in a mutualistic relationship in the vaginal anaerobic environment, producing various antimicrobial compounds like lactic acid, hydrogen peroxide, and bacteriocins that offer protection against potential pathogenic organisms such those causing urinary tract infections, bacterial vaginosis, and candida infections [325,326]. Bacterial vaginosis (BV) is a lower genital tract disorder, highly prevalent in women of reproductive age. It is characterized by a shift in vaginal microbiota with a loss of Lactobacillus species and a substantial increase in the concentration of other microbes such as Gardnerella, Prevotella, Atopobium, Mobiluncus, Bifidobacterium, Sneathia, Leptotrichia, and some novel bacteria in Clostridiales order [325,326]. The current treatment of BV is based on the use of antibiotics; however, the remission is usually temporary and many patients related recurrence after the antibiotic-based treatment [326]. The use of nano-based formulations for vaginal drug delivery of steroids, peptides, antibacterial, antifungal, and antiviral drugs has gained much interest because they offer a sustained and controlled release of the drugs, protect drugs from degradation, increase drug solubilization, improve bioavailability, reduce toxicity, enhance immune modulation, and provide a target-specific drug delivery [327,328]. Also, in vaginal drug delivery, there are some obstacles that need to be overcome, like the low retention time due to vaginal self-cleaning mechanisms and the existence of a mucous barrier that has to be penetrated, clarifying the potential use of nanocarriers and nanofibers for an efficiency delivery of active molecules to vaginal tissues [327]. In a study developed by Krogstad and coworkers, PVA and PVP nanofibers were produced through electrospinning, yielding nanofibers of ≈248 nm (PVA) and ≈297 nm (PVP) (TEM results) in diameter. Then, PEGylated PLGA NPs (via nanoprecipitation with ≈172 nm of diameter (TEM results) were blended with the PVA and PVP solutions prior to electrospinning. In vitro testing showed an >85% cumulative NPs release in less than 30 min., which can be attributed to not fully dissolved PVP solution. In vivo testing revealed that there was a notable increase in the fluorescent signal in cervicovaginal mucus and vaginal tissue in C57/Bl6 mice in the case of topical application of the PVA/PVP-loaded NPs compared to application of the aqueous suspension of NPs. Moreover, there was an improvement in the pharmacokinetic profile of etravirine due to the sustained release of the drug. This study proved that the incorporation of PEGylated PLGA NPs into PVA/PVP electrospun nanofibers enhanced retention time in the vaginal tract [329]. PVP nanofibers were fabricated by electrospinning with ≈557 nm in diameter (TEM results). Then, benzydamine (non-steroidal anti-inflammatory and antiseptic drug)-loaded CSNPs (produced by ionic gelation method with an average particle size varying between 184 nm and 710 nm (DLS results) were blended in the PVP solution prior to electrospinning. Indeed, loaded nanofibers exhibit appropriate tensile strength and contact angles showing that nanofiber formulations on the mucous layer can be completely wetted and release the drug with fast onset. In vitro studies revealed a slower release rate of the loaded nanofibers (53.03% in 24 h, and 59.66% after 48 h). These findings suggest that NP-loaded nanofibers could be an excellent approach for enhanced vaginal drug delivery applications due to their suitable permeability and simple preparation [330].

**Figure 1:**
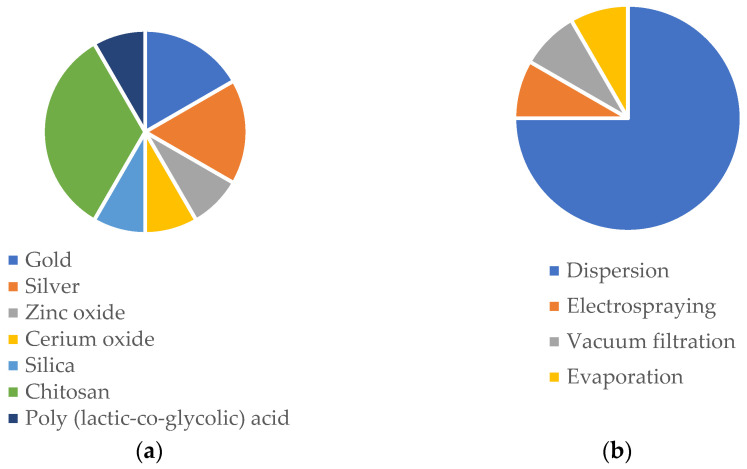

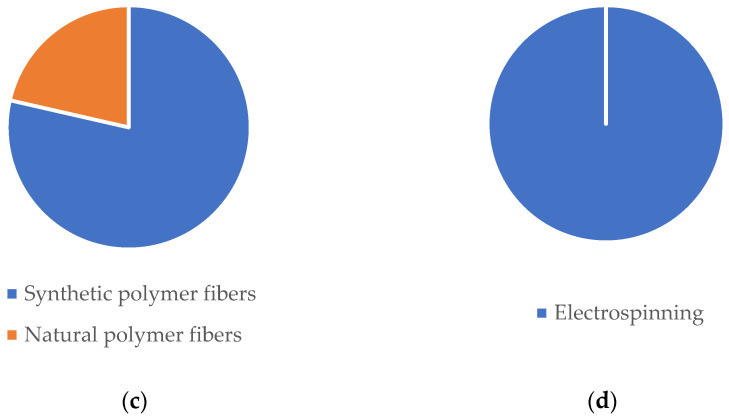
Representative frequency analysis of (**a**) NP composition; (**b**) NP loading on fibers; (**c**) fibers class materials; (**d**) fiber production method for microbial balance applications according to published literature since 2017 (databases: Scopus and PubMed).

Figure 1 illustrates the trends of the last 6 years in the fabrication of fibers functionalized with NPs, including the most commonly used materials, loading strategies, and production methods in microbial balance approaches. CS-based NPs are the major contributors to these numbers followed by inorganic NPs, like silver and gold. Regarding the NP-loading, the dispersion method, in which the NP solution is dissolved in the polymeric solution until a homogenous solution is achieved, is the most explored method. Moreover, synthetic polymers are the most employed to produce fibers in this case, as well as electrospinning as the production method of fibers.

### 3.2. Tissue Regeneration

As a part of a development or repair process, tissue formation is a very complex approach whereupon cell populations self-assemble into functional units [331]. The replication of these events outside the body has gained considerable attention, which has accelerated since the demonstration of the engineering of cartilage and skin [331]. Nowadays, there is worldwide investigation towards an in vitro regeneration of several complex tissues including bone, liver, nerve, blood vessels, among others, focusing on the necessity to provide signals to cell populations in order to promote cell differentiation and proliferation [331]. However, tissue engineering methods face some obstacles such as lack of appropriate biomaterials, unstable and ineffective production of growth factors to stimulate cell communication and adequate response, and ineffective cell growth [332]. NPs are in the front line to help combat those obstacles that tissue engineering is facing. They have some advantages such as their small size, large surface to volume ratio, easy diffusion across biological membranes, as well as a facilitated cell uptake, having the ability to mimic the natural nanometer size scale of extracellular matrix (ECM) components of tissues themselves [332]. Also, the high surface area to volume ratio of nanofibers combined with their porous structure, favors cell adhesion, proliferation, migration, and differentiation, all desired properties for tissue engineering applications [333]. In a study conducted by Shevach et al. spun PCL/gelatin nanofiber scaffolds were produced by electrospinning yielding nanofibers of ≈250 nm in diameter. Then, AuNPs were evaporated on the surface of the produced fibers, creating nanocomposites with a nominal gold thickness of 2.4 and 14 mm. In vivo studies, conducted in cardiac cells isolated from neonatal ventricle myocytes of 1-to-3-day old Sprague–Dawley rats seeded by a single droplet of the developed scaffolds, showed that on day seven cells cultured on AuNPs-containing scaffolds were elongated with massive striation. Moreover, cells cultivated on the 14 mm AuNPs-containing scaffolds were aligned, exhibiting the typical morphology of native heart bundles. In this study the researchers also assessed the performance of the engineered tissues by evaluating the contraction amplitudes of the cell constructs. On day three, high contraction amplitudes were observed in all AuNPs cell constructs [222]. Xi and coworkers developed a functional pH-responsive immunoregulative and neurogenic to treat acute spinal cord injury. Amino-modified PLA oriented micro-sol fibers, containing nerve growth factor (NGF), were produced trough electrospinning with an average fiber diameter of 500 nm (TEM results)). Then, aldehyde cationic liposomes loaded with IL-4 plasmid, produced by reverse evaporation with an average diameter ranging from 70 to 280 nm (DLS results), were grafted by Schiff base bond in the electrospun fibers, which is a type of bond that should break when the pH becomes acidic. In vitro studies showed good mechanical properties, an encapsulation efficacy of 75.77% of the IL-4 plasmid. In vivo studies using SD rats demonstrated that immunoregulatory fiber bundle implantation could reduce the risk of further damage to motor neurons since it successfully inhibited the acute inflammatory response of spinal cord injury as well as encouraged nerve repair [334]. In a recent study, PLLA/Ag composite fiber was produced trough electrospinning, yielding nanofibers of ≈667.92 nm in diameter. AgNPs were uniformly distributed on the inner surface of PLLA fibers. Then, dopamine was self-polymerized on the composite fiber surface to construct the adhesive polydopamine (PDA) film and CS was used to coat AgNPs achieving the steady and slow release of AgNPs. In vitro studies revealed an adequate Ag^+^ release profile (0.2 mg.L^−^^1^ on the 7th day, which is the standard concentration of Ag^+^ in human blood and 0.25 mg.L^−1^ on the 11th day), antibacterial efficiency (100% of antibacterial rate against *E. coli* and *S. aureus*), and excellent angiogenesis performance in vascular endothelial cells (VECs) [335].

**Figure 2:**
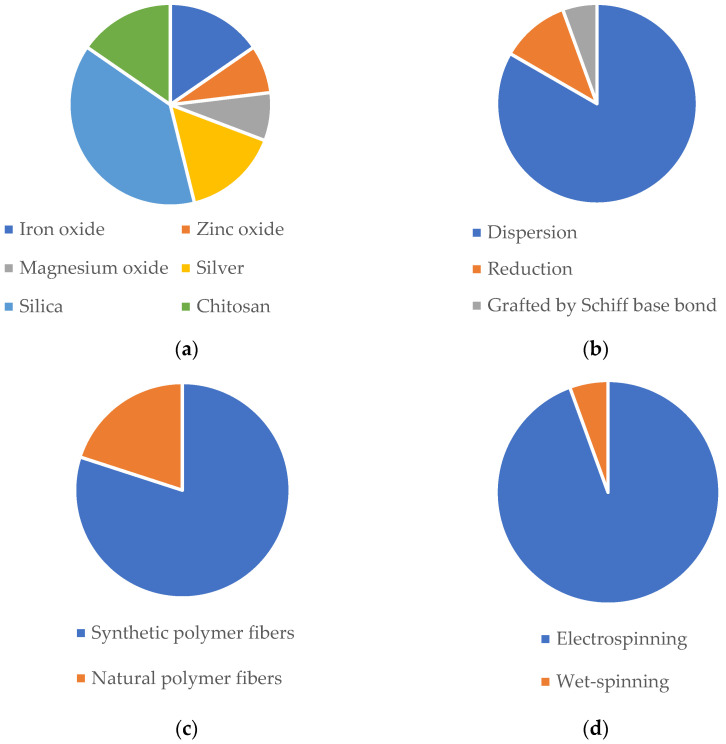
Representative frequency analysis of (**a**) NP composition; (**b**) NP loading on fibers; (**c**) fibers class materials; (**d**) fiber production method for tissue regeneration applications according to published literature since 2017 (databases: Scopus and PubMed).

Figure 2 illustrates the trends of the last 6 years in the fabrication of fibers functionalized with NPs, including the most commonly used materials, loading strategies, and production methods in tissue regeneration approaches. Silica NPs are the major contributors to these numbers followed by inorganic NPs, like silver and iron oxide. It can be observed that dispersion is the most employed method to load NPs, consisting of the dissolution of the NP solution in the polymeric solution until a homogeneous solution is achieved. Moreover, synthetic polymers are the most employed to produce fibers in this case, as well as electrospinning as the production technique.

### 3.3. Anticancer Approaches

Cancer is currently the second leading cause of death worldwide, accounting for nearly 10 million in 2020 according to studies carried out by the World Health Organization (WHO) [336]. The current treatment modalities of cancer are radiation therapy, surgery, and systemic chemotherapy; however, they have several weaknesses, including poor drug uptake, toxicity to the normal cells, insufficient therapeutic efficiency, difficulty in targeting the delivery of drugs to tumor sites, which hampers their efficacy in clinical use [337,338]. NPs can be incorporated in polymer-based fibers to serve as additional carriers to protect the anticancer therapeutic, decrease the occurrence and severity of its side effects, control its release profile, and deliver it to the target cells, maximizing its effect [339]. Moreover, fibers can provide a platform for a controlled and sustained release of the drug at the desired site of action with improved efficacy [340]. In a study conducted by Li et al., palitaxel (PTX), a natural fat-soluble broad spectrum anticancer drug [341] was loaded into lignin NPs (PLNPs—produced via dissolution in tetrahydrofuran, followed by a dialysis process). Then, the produced PLNPs were encapsulated into the polymer solution of PVA/PVP prior to electrospinning, yielding nanofibers of ≈ 207 nm in diameter, to achieve an effective cervical cancer cell inhibition. The obtained PLNPs exhibit a particle size of ≈72 nm, a drug loading rate of ≈8.1%, and an encapsulation efficiency of ≈ 89% of the anticancer drug PTX. The fabricated composite nanofibrous membrane exhibited good particle distribution, thermal stability, mechanical properties, and biocompatibility. In vitro experiments showed that combining lignin NPs by electrospinning not only improved the drug release profile, but also enhanced the hydrophilicity of nanofibrous membranes, which is beneficial to cell adhesion and proliferation. Also, PVA/PVP 2% PLNPs nanofibrous membrane showed good cell inhibition capability (HeLa cells survival rate of ≈21% at day 7), indicating that the addition of PLNPs can effectively inhibit cancer cells for a long time. Laser confocal microscopy was used to investigate the morphology of HeLa cells demonstrating an apoptosis process in cell number and cytoplasmic vacuolation, indicating that PVA/PVP-PLNP membranes exhibit a long-term effective anticancer ability [342]. In another study, a PCL/GN nanofiber was used for co-encapsulation of free-curcumin (CUR) and CUR-loaded mesoporous silica NPs (CUR@MSNs) through electrospinning, resulting in a novel drug-loaded nanofibrous scaffold—CUR/CUR@MSNs-NFs with enhanced anticancer efficacy. Firstly, MSNs NPs were produced (via a modified Stöber method), followed by CUR encapsulation (CUR@MSNs). Then, the produced NPs were blended in a PCL/GN solution prior to electrospinning, yielding nanofibers of 610 nm in diameter. The produced CUR@MSN NPs exhibited a size of 117 nm, a zeta potential of + 3.3 mV, and a drug loading content of 24.4%. The release pattern of CUR/CUR@MSNs-NFs was characterized to have an initial burst release of 54% in the first 3 days, culminating in a late sustained release of about 100% of loaded CUR over 5 weeks. Also, CUR/CUR@MSNs-NFs exhibited higher toxicity towards MDA-MB-231 breast cancer cells after a period of 72 h of incubation time, significantly more anti-migratory effect, a more pronounced effect in apoptosis induction and reduction of the cell number, showing the greatest decrease for Bcl-2, which suggested that the two-stage CUR discharge from CUR/CUR@MSNs-NFs promoted cell apoptosis and the anticancer efficiency of CUR through effective modifications in the expression of the genes associated with the programmed cell death. Interestingly, the appropriate viability and lack of significant toxicity observed in the human normal breast MCF-10a cells treated with the CUR/CUR@MSNs-NFs disclosed its potential in specifically targeting the breast cancer cells with minimal intoxication to normal breast cells [343].

In another study, siRNA (36 nM) was loaded into Holo transferrin PEG-liposomes (produced via evaporation followed by a consecutive series of vortexing and heating). Then, the produced liposomes were blended with the polymer solution of PCL/GN prior to the electrospinning, to achieve a targeted cancer therapy. The obtained liposomes exhibited a spherical shape with a particle size of 100 nm (TEM results) and 117.2 nm (DLS results), a zeta potential of −11 mV and an siRNA loading efficiency of 92.3%. In vitro studies in HUVEC (human endothelial cells) and K562 (lymphoblasts isolated from the bone marrow of a 53-year-old chronic myelogenous leukemia patient) cells co-culture showed that liposomes exhibited 3:1 specificity between cancerous K562 cells in relation to healthy HUVEC, and the combination of controlled release of resveratrol and targeted liposomes significantly affected K562 cell apoptosis over 8 days. Also, this combination therapy is as effective in the presence and absence of HUVEC, indicating that this can be a promising target cancer therapy [344].

**Figure 3:**
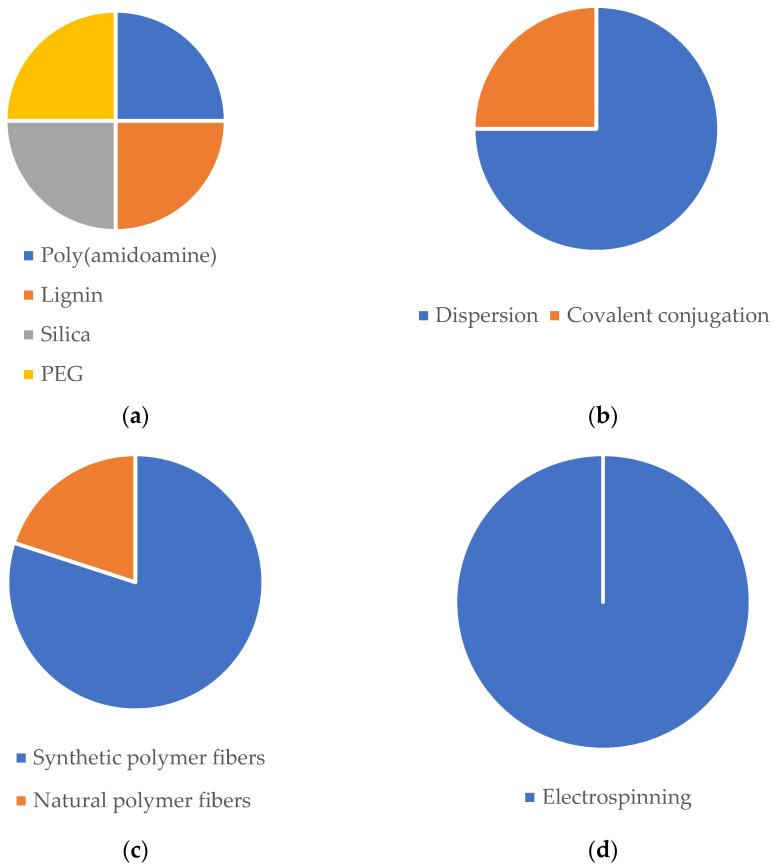
Representative frequency analysis of (**a**) NP composition; (**b**) NP loading on fibers; (**c**) fibers class materials; (**d**) fiber production method for anticancer approaches according to published literature since 2017 (databases: Scopus and PubMed).

Figure 3 illustrates the trends of the last 6 years in the fabrication of fibers functionalized with NPs, including the most commonly used materials, loading strategies and production methods in anticancer approaches. Silica, lignin, PEG, and poly(amidoamine) NPs are the contributors to these numbers. Regarding the NP loading, the dispersion method, in which the NP solution is dissolved in the polymeric solution until a homogenous solution is achieved is the most explored method, followed by covalent conjugation. Synthetic polymers are the most used to produce fibers and electrospinning as the production method of fibers.

**Table 2:** Intended application of NP-loaded polymer-based fiber.

NP			NP-Loaded Fibers								
Composition	Production	Characteristics	Composition	Production Method	NP Loading	Characteristics	Architecture	Bioactivity	Administration	Intended Biomedical Effect	Ref.
Au/mercaptophenylboronic acid	One-pot method	Spherical; d_TEM_ = 1.8 nm; ζ = −5.55 mV	PCL/GN	Electrospinning	Dispersion (solubilization of NP within the polymeric solution)	Bead-free; d_TEM_ = 560 nm	Nanofibrous mat	Improved antibacterial efficiency against *S. aureus* and MDR *S. aureus*. Non-toxic towards HUVECs and NIH 3T3 cells. No hemolysis in rat blood. 89% and 98% of mice wound closure in 14 days, both with *S. aureus* and MDR *S. aureus* infection.	Topical	Microbial balance	[222]
Ag and CS; Phenytoin	Reduction method	Spherical; d_DLS_ = 53.6 nm; d_TEM_ = 30 nm; ζ = +48 mV	PCL/PVA	Electrospinning	Dispersion (solubilization of NP within the polymeric solution)	Bead-free; d_TEM_ = 368 nm	Coaxial nanofibrous mat	Slowly and steady release of phenytoin (16.7% in 6h and 53.8% in 7 days). Antibacterial efficiency against *S. aureus* and *E. coli*. Survival and proliferation of 3T3 cells. The scaffold demonstrated the ability to swell to absorb wound exudates.	Topical	Microbial balance	[345]
ZnO; oregano essential oil	-	Commercially acquired with size ≤ 40 nm	PLCL	Electrospinning	Dispersion (solubilization of NP within the polymeric solution)	Bead-free; d_TEM_ = 1.04 µm	Core-shell nanofibrous mat	Antioxidant potency. Antibacterial efficiency against *E. coli* and *S. aureus*. Survival and proliferation of 3T3 cells. In vivo studies revealed 89.7% diabetic rats wound closure in 15 days without bacterial infections.	Topical	Microbial balance	[324]
Cerium oxide	Redox chemistry	Quasi-spherical; d_TEM_ = 42 nm; ζ = +30.8 mV	PCL/GN	Electrospinning	Dispersion (solubilization of NP within the polymeric solution)	Bead-free; d_SEM_ = 486 nm	Nanofibrous mesh	Proliferation of 3T3 cells. Antioxidant properties	Topical	Microbial balance	[4]
Zinc doped hollow mesoporous silica nanospheres; Ciprofloxacin	Sol-gel method	Spherical; d_TEM_ = 100 nm	PCL	Electrospinning	Dispersion (solubilization of NP within the polymeric solution)	Bead-free; d_SEM_ = 2 µm	Nanofibrous mat	Antibacterial activity against *E. coli*. No cytotoxic effects on HUVECs and HDFs. After 13 days, healthy tissue appeared in the wound area of *E. coli*-infected mice.	Topical	Microbial balance	[346]
Carboxymethyl CS; Antimicrobial peptide:OH-30	Electrostatic droplet	Spherical; d_TEM_ = 164.6 nm; ζ = −37.6 mV	PVA/CS	Electrospinning	Dispersion (solubilization of NP within the polymeric solution)	Bead-free; d_SEM_ = 327 nm	Nanofibrous mat	Cumulate release of the OH-CATH30 around 66% in 24 h. Antibacterial efficiency against *E. coli* and *S. aureus*. No cytotoxic effects towards HaCaT cells. Around 98% of mice wound closure in 12 days.	Topical	Microbial balance	[323]
CS; TPP; Curcumin	Ionic gelation	d_TEM_ = 32.17 nm	PCL/CS/Curcumin	Electrospinning	Electrospraying	Bead-free; d_SEM_ = 99.84 nm	Nanofibrous mat	Slow and sustained release of curcumin of 67.2% in 6 days. Antioxidant activity. Antibacterial activity against MRSA and *E. coli* (ESBL). Proliferation and survival of HDF cells. 98.5% wound closure of MRSA-infected mice wounds.	Topical	Microbial balance	[347]
CS; TPP; Curcumin	Ionic gelation	Spherical; d_TEM_ = 359 nm; ζ = −10.7 mV	PCL/GN	Electrospinning	Dispersion (solubilization of NP within the polymeric solution)	Bead-free; d_SEM_ = 1548 µm	Nanofibrous mat	Good mechanical properties and swelling capacity. Accumulate release of curcumin of 23% in 6 h. Cytocompatibility towards EnSCs cells. In vivo studies showed 73.4% of wound closure in 14 days.	Topical	Microbial balance	[348]
Ag	-	Commercially acquired with size of 15 nm	PLA/Cellulose nanofibrils	Electrospinning	Vacuum filtration (Ag NPs suspension was filtrated for the PLA nanofibers)	Bead-free; d_FESEM_ = 1.44 µm	Nanofibrous mat	Good tensile strength and hydrophilic mats. Biocompatibility towards CjECS and CECs ocular epithelial cells. Antibacterial efficiency against *S. aureus* and *E. coli*.	Transdermal	Microbial balance	[349]
PEGylated PLGA; Etravirine	Nanoprecipitation	d_TEM_ = 172 nm	PVA and PVP	Electrospinning	Dispersion (solubilization of NP within the polymeric solution)	Bead-free; d_TEM_ (PVA) = 248 nm; d_TEM_ (PVP) = 297 nm	Nanofibrous mat	Increase in the fluorescent signal in cervicovaginal mucus and vaginal tissue in C57/Bl6 mice in the case of topical application of the PVA/PVP-loaded NPs. Improvement in the pharmacokinetic profile of etravirine due to the sustained release of the drug.	Transdermal	Microbial balance	[329]
CS; Benzydamine	Ionic gelation	d_DLS_ varying between 184 and 710 nm	PVP	Electrospinning	Dispersion (solubilization of NP within the polymeric solution)	Bead-free; d_TEM_ = 557 nm	Nanofibrous mat	Appropriate tensile strength and contact angles. 53.03% of drug release in 24 h and 59.66% after 48 h.	Transdermal	Microbial balance	[330]
Au	-	d_TEM_ = 10 nm	PCL/GN	Electrospinning	Evaporation of gold NPs (the functional groups of gelatin were the binding sites for the evaporated NPs)	Bead-free; d_TEM_ = 260 nm	Nanofibrous mat	Differentiation, growth, and maturation of neurons. Elaborated neuronal growth and axonal elongation, leading to more complex neuronal networks	Transdermal	Microbial balance	[14]
Ag	-	-	PLLA	Electrospinning	In situ reduction method (PLLA nanofibers immersed in silver nitrate, washed, and dried)	Bead-free; XRD patterns at 38.26°, 44.37° and 76.61°	Nanofibrous mat	Antibacterial activity against *E. coli* and *S. aureus*. Biocompatibility towards MC3T3 and L929 cells.	Topical	Tissue regeneration	[350]
Ag; CS	-	-	PLLA	Electrospinning	In situ reduction method (PLLA nanofibers immersed in silver nitrate, washed, and dried)	Bead-free; d_TEM_ = 667.92 nm	Nanofibrous mat	Slow and steady release of Ag NPs (0.2 mg/L on day 7 and 0.25 mg/L on day 11). Antibacterial efficiency against *E. coli* and *S. aureus*. Excellent angiogenesis performance in VECs cells.	Topical	Tissue regeneration	[335]
Iron oxide (SPIONs); Casein	Ultrasonication	Spherical; d_SEM_ = 36 nm	Silk-fibroin	Electrospinning	Dispersion (solubilization of NP within the polymeric solution)	Bead-free; d_SEM_ = 251.78 nm	Nanofibrous mat	Good mechanical properties. Biocompatibility towards ECCs. Survival and proliferation of ECCs.	Transdermal	Tissue regeneration	[351]
ZnO	-	Commercially acquired with size ranging between 10 and 30 nm	Outer layers: PVA, chitosan and shell protein; Middle layer: PEO, GN and ZnONPs	Electrospinning	Dispersion (solubilization of NP within the polymeric solution)	Bead-free; d_SEM_ = 108, 128.5, 138.5, 140, 153.7 nm	Tri-layer nanofibrous composite	Good mechanical properties and swelling reduction of three-layer nanofibers with incorporation of NPs. Accelerated proliferation of fibroblast cells.	Transdermal	Tissue regeneration	[352]
Iron oxide (SPIONs)	-	d_TEM_ = 11–12 nm	PLLA	Electrospinning	Dispersion (solubilization of NP within the polymeric solution)	Bead-free; d_TEM_ = 1.73, 1.65, 1.96, 1.76, 2.03 µm	Nanofibrous mat	In vivo studies showed that neurons yielded a significant increase in the mean neurite outgrowth. Cytocompatibility towards neurons cells.	Intravenous injection	Tissue regeneration	[353]
MgO	Hydroxide precipitation and sol-gel	Hexagonal and cubical shape; d_TEM_ = 40–60 nm	PCL	Electrospinning	Dispersion (solubilization of NP within the polymeric solution)	Bead-free; d_SEM_ = 0.2–0.6 µm	Nanofibrous mat	Improved mechanical properties, promotion of adhesion, proliferation, and differentiation of MG-63 cells. In vivo studies revealed good biocompatibility with an initial moderate inflammatory response near the implant site which became less intense at eighth week.	Subcutaneous implant	Tissue regeneration	[354]
Calcium phosphate	-	-	PLGA	Electrospinning	Dispersion (solubilization of NP within the polymeric solution)	Bead-free; d_SEM_ = 810 nm	Nanofibrous mat	Good biocompatibility towards rADSCs cells. Thermal treatment of NPs improved in vitro mineralization properties of nanofibers. The presence of NPs resulted in higher elasticity and ductility of nanofibers.	Transdermal	Tissue regeneration	[355]
Mesoporous silica; Paclitaxel; Endothelial growth factor (VEGF)	Stöber method	Pore size SEM = 3.17 nm	PLA	Electrospinning	Dispersion (solubilization of NP within the polymeric solution)	Bead-free; d_TEM_ = 1.26 µm	Nanofibrous mat	Promoted endothelial cell proliferation of HUVECs, inhibiting the proliferation of SMCs. In vivo studies revealed improved immediate and mid-term complete aneurysm occlusion rates, earlier endothelialization promotion and better lumen restenosis.	Transdermal	Tissue regeneration	[356]
Mesoporous silica; Dexamethasone	Surfactant templating	Spherical; d_TEM_ = 100–200 nm	PLGA/GN	Electrospinning	Dispersion (solubilization of NP within the polymeric solution)	Bead-free; Average thickness of 0.088 mm and 0.305 mm	Bi-layer nanofibrous membrane	Good mechanical properties. Sustained release of dexamethasone (38.8% after 21 days). Proliferation of L929 cells and enhanced osteoinductive capacity. Antibacterial activity against *E. coli* and *S. aureus*.	Transdermal	Tissue regeneration	[357]
Mesoporous silica	Template removal	Spherical; d_TEM_ = 70.9 nm	PLGA and PLGA/GN	Electrospinning	Dispersion (solubilization of NP within the polymeric solution)	Bead-free; d_TEM_ (PLGA + NPs) = 418 nm; d_TEM_ (PLGA/gelatin + NPs) = 267 nm	Nanofibrous mat	Enhanced hydrophilicity and tensile mechanical properties of scaffold upon incorporation of NPs and gelatin. Improved cell attachment and proliferation of PC12 cells.	Transdermal	Tissue regeneration	[358]
Mesoporous silica	Sol-gel method	-	PLA/PANI	Electrospinning	Dispersion (solubilization of NP within the polymeric solution)	Bead-free; d_SEM_ = 150–300 nm	Nanofibrous mat	Biocompatibility towards C2C12 myoblasts. Controlled release of NPs from the scaffold. Promoted tissue vascularization on chicken embryo chorioallantoic membrane.	Transdermal	Tissue regeneration	[359]
Aldehyde cationic liposomes; IL-4 plasmid	Reverse evaporation method	d_DLS_ varying between 70 and 280 nm	PLA/NGF	Electrospinning	Grafted by Schiff base bond	Bead-free; d_TEM_ = 500 nm	Nanofibrous mat	Good mechanical properties. In vivo studies revealed reduced risk of further damage to motor neurons since it successfully inhibited the acute inflammatory response of spinal cord injury and encouraged nerve repair.	Transdermal	Tissue regeneration	[334]
Dextran glassy; bFGF	-	d_SEM_ = 200 to 500 nm	PLLA	Electrospinning	Dispersion (solubilization of NP within the polymeric solution)	Bead-free; d_SEM_ = 0.27 µm	Nanofibrous mat	Encapsulation efficiency of 67.03% and no burst release and a controlled release kinetic of nearly 30 days. Promotion of cell adhesion and proliferation of C3 cells. Significantly increased tendon thickness in mice after 21 days.	Transdermal	Tissue regeneration	[360]
CS; Veratric acid	Ionic gelation	Spherical; d_TEM_ = 99 nm	PCL (core)/PVP (sheath)	Electrospinning	Dispersion (solubilization of NP within the polymeric solution)	Bead-free; d_TEM_ = 515 nm	Coaxial nanofibrous mat	Good mechanical properties and protein adsorption. Mineralization capacity. Controlled release of veratric acid (60% release in 20 days). Biocompatibility towards mMSCs cells, and osteoblastic differentiation.	Transdermal	Tissue regeneration	[361]
CS; Nell-1 growth factor	Ionic gelation	Spherical; d_TEM_ = 207 nm	PLLA-CL (core)/Collagen I (sheath)	Electrospinning	Dispersion (solubilization of NP within the polymeric solution)	Bead-free; d_TEM_ = 5 to 50 µm	Coaxial nanofibrous mat	Bioactivity of Nell-1 towards sao-2 cells release from the NPs-loaded scaffold was increased. hBMSCs showed elongated morphology and alignment when cultured with the NP-loaded scaffold. In vitro studies showed that Nell-1 released from the NP-loaded scaffold significantly increased the GAG content (component of hyaline cartilage.	Transdermal	Tissue regeneration	[362]
PCL; PLGA; Ciprofloxacin	Nanoprecipitation	d_DLS_ = 250 nm	PEOT/PBT	Electrospinning	Dispersion (solubilization of NP within the polymeric solution)	*-*	Nanofibrous mat	No cytotoxicity towards HaCaT and hMSCs cells. Antibacterial activity against *S. aureus* and *P. aeruginosa*. In vitro studies showed that all ciprofloxacin-loaded NPs were able to hamper *S. aureus* adhesion and invasion to HaCaT cells as well as for *P. aeruginosa.*	Transdermal	Tissue regeneration	[363]
Titanium nitride	Laser ablation	-	PCL	Electrospinning	Dispersion (solubilization of NP within the polymeric solution)	Bead-free; d_SEM_ = 0.403 and 1.1 µm	Nanofibrous mat	Thermal analysis demonstrated that the incubation of TiN NPs in nanofibers led to slight variations in mass degradation initiation and phase behavior. In vitro studies revealed biocompatibility towards 3T3 fibroblast cell.	Transdermal	Tissue regeneration	[364]
Silica	Direct self-assembly	-	Cellulose	Wet-spinning	Dispersion (solubilization of NP within the coagulation bath)	-	Fibers	The incorporation of silica NPs resulted for all types of fibers in an enhancement of the strength and superior toughness.	Transdermal	Tissue regeneration	[365]
Holo-transferrin conjugated liposomes; SiRNA (36 nM)	-	Spherical; d_TEM_ = 100 nm; d_DLS_ = 117.2 nm; ζ = −11 mV	PCL/GN	Electrospinning	Dispersion (solubilization of NP within the polymeric solution)	-	Microfibrous mat	Produced liposomes showed 3:1 specificity between cancerous K562 cells in relation to healthy HUVEC. In vitro studies showed inhibition of sphingosine kinase 1 in K562 cells.	Transdermal	Anticancer approaches	[344]
Amine-terminated generation 5 poly(amidoamine) dendrimers	-	-	Cellulose Acetate assembled layer-by-layer with a bilayer of PDADMAC and PAA	Electrospinning	Covalent conjugation (via the 1-ethyl-3-(3-dimethylaminopropyl) carbodiimide hydrochloride coupling reaction)	Bead-free; d_SEM_ = 431.6 nm	Sandwich	Cell capture efficiencies of 36.3% and 82.7% at 40 and 60 min., respectively, in KB-HFAR cells. In vitro studies showed that the developed mat displays specificity to capture FAR-overexpressing cancer cells via ligand-receptor interactions.	Transdermal	Anticancer approaches	[366]
Lignin; Paclitaxel	Dissolution in tetrahydrofuran, followed by a dialysis process	Spherical; d_TEM_ = 72 nm	PVA/PVP	Electrospinning	Dispersion (solubilization of NP within the polymeric solution)	Bead-free; d_TEM_ = 207 nm	Nanofibrous mat	Good thermal stability, mechanical properties, and biocompatibility towards HeLa cells with a survival rate of 21% at day 7. exhibited a long-term effective anticancer ability by promoting an apoptosis process in cell number and cytoplasmic vacuolation.	Transdermal	Anticancer approaches	[342]
Mesoporous silica; Curcumin	Modified Stöber method	Spherical; d_DLS_ = 117 nm; ζ = + 3.3 mV	PCL/GN/Curcumin	Electrospinning	Dispersion (solubilization of NP within the polymeric solution)	Bead-free; d_TEM_ = 610 nm	Nanofibrous mat	Exhibited higher toxicity towards MDA-MB-231 breast cancer cells after a period of 72 hr. incubation time, significantly more anti-migratory effect, a more pronounced effect on apoptosis induction, and reduction of the cell number and showed the greatest decrease for Bcl-2, suggesting that the two-stage curcumin discharge from the scaffold promoted cell apoptosis.	Transdermal	Anticancer approaches	[343]

Au: gold; PCL: polycaprolactone; GN: gelatin; TEM: transmission electron microscopy; MDR: multidrug-resistant; HUVECs: human umbilical vein endothelial cells; NIH 3T3: fibroblast cell line; Ag: silver; CS: chitosan; DLS: dynamic light scattering; PVA: poly(vinyl alcohol); ZnO: zinc oxide; PLCL: poly(lactide-co-epsilon-caprolactone); SEM: scanning electron microscopy; HDFs: human dermal fibroblasts; HaCaT: immortalized human keratinocytes; TPP: thiamine pyrophosphate; MRSA: methilicin-resistant *S. aureus*; EnSCs: embryonic stem cells; FESEM: field emission scanning electron microscopy; PLA: polylactic acid; CECs: circulating endothelial cells; PEG: polyethylene glycol; PLGA: poly(lactic-co-glycolic acid); PVP: polyvinylpyrrolidone; PLLA: poly(lactic acid); XRD: X-ray powder diffraction; L929: mouse fibroblast cell line; VECs: vascular endothelial cells; ECCs: embryonal carcinoma cells; PEO: polyethylene glycol; MgO: magnesium oxide; MG-63: human osteoblastic line; rADSCs: adipose-derived stem cells; VEGF: vascular endothelial growth factor; SMCs: smooth muscle cells; PC12: clonal cell line derived from a pheochromocytoma of the rat adrenal medulla; PANI: polyaniline; C2C12: myoblast cell line; NGF: nerve growth factor; bFGF: fibroblast growth factor 2; mMSCs: MM cancer stem cells; hBMSCs: bone-marrow-derived mesenchymal stem cells; PEOT/PBT: poly(butylene terephthalate); K562: lymphoblast cells; PDADMA: poly(diallyldimethylammonium chloride); PAA: poly (acrylic acid); HeLa: cervical cancer cells.

## 4. Conclusions

It is well known that NPs have a very important role in the evolution of therapeutics since they present outstanding surface properties that allow an improved effect when compared with bulky traditional additives and materials. NP drug delivery systems have the potential to improve the current disease therapy due to their ability to deliver drugs in the optimum dosage range often resulting in increased therapeutic efficiency, reduced side effects, and improved patient compliance. Two important drawbacks regarding NPs’ application are their rapid clearance of circulation during systemic delivery and their instability in biological environments, which are caused by interactions with biological barriers and tunable NP parameters, such as composition, size, surface modifications, core properties, and targeting ligand functionalization. The development of hybrid composite scaffolds, which are able to maximize the biological effects of NPs, minimizing their associated drawbacks in biomedical applications, is something to be sought. One important strategy consists in incorporating NPs into/onto polymer-based electrospun nanofibers, as these are ideal local delivery carriers with high porosity that can be tuned in diameter to influence cell attachment, proliferation, migration, and differentiation. On another hand, fiber-based scaffolds functionalized with NPs are gaining much attention in tissue engineering, biomedicine, and controlled drug delivery. These can serve as platforms to achieve a modulate localized and controlled delivery of the intended therapeutic agents. Fibers reinforced with NPs with adequate biocompatibility and biodegradability present usefulness for tissue engineering and drug delivery/pharmaceuticals applications. Overall, this topic has been an object of great attention from the research community since the results were found highly promising. Still, the reduced number of in vivo studies in humans continues to be one of the major obstacles that needs to be overcome. It seems of critical importance to apply all the efforts to successfully investigate the biocompatibility and effectiveness of these hybrid composite scaffolds in humans. Thus, most of the studies reviewed in this article must be further investigated before products are ready for commercialization and to be applied in clinical environments.

## Figures and Tables

**Table 1:** Summarized advantages and limitations of the inorganic and organic nanoparticles most commonly functionalized and integrated into polymer-based fibers.

		Type	Advantages	Limitations
Nanoparticles	Inorganic	Silver 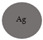	High-scale production; Long-term stability; Antimicrobial properties.	Limited drug loading capacity; Associated toxicity; Limited capacity to load lipophobic drugs.
		Gold 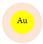	Simplicity; High stability; Low resistivity.	Low solubility; Short half-life; Associated toxicity.
		Iron oxide 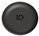	Low toxicity; Colloidal stability; High magnetic susceptibility.	Agglomeration Limited therapeutic efficacy; Non-scalable.
		Zinc oxide 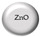	High solubility Antibacterial efficacy; Low-cost.	Associated toxicity.
		Magnesium oxide 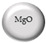	High chemical stability; Low toxicity; High electrical permitivity.	Ecotoxicity.
		Cerium oxide 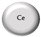	Antioxidant properties; High chemical stability; High ionic conductivity.	Associated toxicity.
		Titanium dioxide 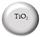	Antimicrobial properties; Photo-catalytic properties.	Associated toxicity.
		Silica 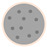	Large surface area; Low toxicity; High hydrophobicity.	Low encapsulation ability; Leakage and inactivation of loading substances; Scattered size distribution.
	Organic	Polymeric micelles 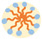	Protection against drug clearance; Ability to load hydrophobic drugs; Controlled release of load drugs.	Reduces payload; Low stability in aqueous medium.
		Chitosan-based 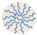	Low toxicity; Versatility; Biodegradability.	Low solubility in neutral and alkaline pH; Low mechanical resistance; Difficulty in controlling pore size.
		Dendrimers 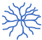	High loading capacity; Bioavailability.	Associated toxicity; Low hydrosolubility.
		Liposomes 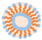	Biocompatibility; Biodegradability; Non-immunogenicity	High production cost; Low solubility; Short half-life.

## Abbreviations

Ag	silver
Au	gold
BDD	boron-doped diamond
bFGF	fibroblast growth factor 2
BV	bacterial vaginosis
C2C12	myoblast cell line
CA	cellulose acetate
Ce	cerium
CECs	circulating endothelial cells
CMC	critical micelle concentration
CS	chitosan
CTAB	cetyltrimethylammonium bromide
CUR	curcumin
DLS	dynamic light scattering
DMAc	dimethylacetamide
DMF	N,N-dimethylformamide
DMSO	dimethylsulfoxide
DOX	doxorubicin
DSS	dioctylsodium dodecyl sulfate
ECCs	embryonal carcinoma cells
ECM	extracellular matrix
ELS	Electrophoretic light scattering
EnSCs	embryonic stem cells
FDA	food and drug administration
Fe	iron
FESEM	field emission scanning electron microscopy
GN	gelatin
GRAS	generally recognized as safe
HaCaT	immortalized human keratinocytes
hBMSCs	Bone-marrow-derived mesenchymal stem cells
HDFs	human dermal fibroblasts
HeLa	cervical cancer cells.
HMSN	hollow mesoporous silica nanoparticles
HR-TEM	high resolution transmission electron microscopy
HUVECs	human umbilical vein endothelial cells
IO	iron oxide
K562	lymphoblast cells
L929	mouse fibroblast cell line
MDR	multidrug-resistant
Mg	magnesium
MG-63	human osteoblastic line
MgO	magnesium oxide
MMP	matrix metallo proteinase
mMSCs	MM cancer stem cells
MRI	magnetic resonance imaging
MRSA	methilicin-resistant *S. aureus*
MSNs	mesoporous silica nanoparticles
MTX	methotrexate
NADH	nicotinamide adenine dinucleotide
NGF	nerve growth factor
NIH 3T3	fibroblast cell line
NPs	nanoparticles
PAA	poly (acrylic acid)
PAN	polyacrylonitrile
PANI	polyaniline
PC12	clonal cell line derived from a pheochromocytoma of the rat adrenal medulla
PCL	polycaprolactone
PDA	polydopamine
PDADMA	poly(diallyldimethylammonium chloride)
PdI	polydispersity index
PDLLA	poly (dl-lactide)
PE	polyethylene
PEG	polyethylene glycol
PEO	polyethylene glycol
PEOT/PBT	poly(butylene terephthalate)
PICsomes	polyion complex vesicles
PLA	polylactic acid
PLCL	poly(lactide-co-epsilon-caprolactone)
PLDA	poly (d-lactide)
PLGA	poly(lactic-co-glycolic acid)
PLLA	poly(lactic acid)
PM	polymeric micelles
PP	polypropylene
PPE	personal protective equipment
PS	polystyrene
PSD	particle-size distribution
PTX	paclitaxel
PU	polyurethane
PVA	poly(vinyl alcohol)
PVP	polyvinylpyrrolidone
rADSCs	adipose-derived stem cells
RGD	Arginylglycylaspartic acid
SDS	sodium dodecyl sulfate
SEM	scanning electron microscopy
Si	silica
SMCs	smooth muscle cells
SSS	sodium silicate solution
Ta	tantalum
TAM	tamoxifen
TEM	transmission electron microscopy
TEOS	tetraethylorthosilicate
THF	tetrahydrofuran
Ti	titanium
TPP	thiamine pyrophosphate
VECs	vascular endothelial cells
VEGF	vascular endothelial growth factor
WHO	world health organization
XRD	X-ray powder diffraction
Zn	zinc
ZnO	zinc oxide
